# COVID-19 public health and social measures (PHSM) and early childhood developmental concerns in Scotland: an interrupted time series analysis

**DOI:** 10.1016/j.lanepe.2025.101525

**Published:** 2025-11-25

**Authors:** Iain Hardie, Louise Marryat, Aja Murray, Josiah King, Kenneth Okelo, Lynda Fenton, Abigail K. Stevely, James P. Boardman, Michael V. Lombardo, Sarah J. Stock, Rachael Wood, Bonnie Auyeung

**Affiliations:** a Department of Psychology, School of Philosophy, Psychology and Language Sciences, University of Edinburgh, Edinburgh, UK; b School of Health Sciences, University of Dundee, Dundee, UK; c Public Health Scotland, Edinburgh and Glasgow, UK; d Sheffield Addictions Research Group, School of Medicine and Population Health, University of Sheffield, Sheffield, UK; e Centre for Clinical Brain Sciences, University of Edinburgh, Edinburgh, UK; f Institute for Regeneration and Repair, University of Edinburgh, Edinburgh, UK; g Laboratory for Autism and Neurodevelopmental Disorders, Center for Neuroscience and Cognitive Systems, Istituto Italiano di Tecnologia, Rovereto, Italy; h Usher Institute, University of Edinburgh, Edinburgh, UK

**Keywords:** COVID-19 Public Health and Social Measures, Child development

## Abstract

**Background:**

COVID-19 public health and social measures (PHSM) may have affected children's development, for example by reducing their interaction with others. We examined associations between PHSM and developmental concerns among young children in Scotland.

**Methods:**

We utilised data from routine 13–15 month and 27–30 month child health reviews, covering all children in Scotland who took part in reviews between January 2019 and August 2023 and had full developmental data. Interrupted time-series analysis assessed slope changes in the weekly proportion of children with health visitor-identified developmental concerns following the March 2020 introduction of, and August 2021 removal of, PHSM. Outcomes were any 13–15 month and 27–30 month developmental concerns, and domain-specific concerns regarding speech-language-communication, problem solving, gross motor, personal-social, emotional-behavioural and fine motor development.

**Findings:**

Weekly proportions were based on 257,532 children, covering 13–15 month review records for 186,265 children (95,506 [51.3%] male and 90,756 [48.7%] female) and 27–30 month review records for 186,766 children (95,209 [51.0%] male and 91,557 [49.0%] female). The March 2020 PHSM introduction was associated with a slope change increase in the proportion of children with any developmental concerns (+0.091 percentage points per week [95% CI: 0.065, 0.116] at 13–15 months and +0.076 percentage points per week [95% CI: 0.048, 0.104] at 27–30 months. The August 2021 PHSM removal was associated with a slope change decrease in the proportion of children with any developmental concerns at 27–30 months (−0.067 percentage points per week [95% CI: −0.088, −0.046]), but not 13–15 months, in the main analysis. Results were broadly consistent across developmental domains.

**Interpretation:**

COVID-19 PHSM were associated with increased developmental concerns among young children in Scotland. While leveraging interrupted time-series analysis yields findings consistent with a causal impact of PHSM, the influence of potential time-varying confounders cannot be ruled out.

**Funding:**

Economic and Social Research Council.

**Research in context**

**Evidence before this study**

We searched PubMed for studies published between 1st March 2020 and 16th April 2025 examining COVID-19 PHSM and child developmental outcomes. For this, we used the search terms ("COVID-19"[Title/Abstract] OR "SARS-CoV-2"[Title/Abstract]) AND "pandemic"[Title/Abstract] AND ("child"[Title/Abstract] OR "children"[Title/Abstract] OR "infant"[Title/Abstract]) AND ("development"[Title/Abstract] OR "developmental"[Title/Abstract] OR "neurodevelopment"[Title/Abstract] OR "neurodevelopmental"[Title/Abstract] OR "ASQ"[Title/Abstract] OR SDQ"[Title/Abstract]).

We identified two relevant review articles, and 29 original research articles quantitatively examining associations between the COVID-19 pandemic, pandemic-related PHSM and child developmental outcomes. This included studies from the United States, Canada, Mexico, Costa Rica, Uruguay, Ethiopia, Bangladesh, China, Japan, South Korea, Ireland, the United Kingdom, Germany, Denmark and Portugal. Most children analysed were aged 0–3 years, but some studies included children up to age 10 years. Whilst some of these studies found no associations between the COVID-19 pandemic, PHSM, and child developmental outcomes, the majority (21 out of 29 studies) highlighted negative developmental outcomes following the start of the pandemic and associated PHSM. Many studies were limited by being local-level studies or having small sample sizes (14 out of 29 studies had a sample size of 1000 children or less). Of the eight largest studies (i.e., those that were population-level or had a sample size of 10,000 or more), six found negative developmental outcomes during the pandemic, whilst two found no significant associations or had mixed findings. We found no studies in Europe that were population-level studies or had a sample size of 10,000 or more and were specifically focussing on the effect of the pandemic or associated PHSM. The review articles we identified noted that there is a paucity of longitudinal studies and that more extensive studies would be able to provide more concrete insights.

**Added value of this study**

This study utilised data from routine child health reviews to assess associations between COVID-19 PHSM and the weekly proportion of children with developmental concerns at age 13–15 months and 27–30 months in Scotland. We analysed data from 257,532 individual children, including 186,265 13–15 month child health review records and 186,766 27–30 month child health review records, making this the largest known analysis of population-level statistics to assess COVID-19 PHSM and child developmental outcomes in the United Kingdom or Europe. Our findings suggest that the introduction of PHSM was associated with increased developmental concerns at both ages. Notably, concerns at 13–15 months continued to rise even after PHSM were lifted, whilst concerns at 27–30 months stopped increasing but remained above pre-pandemic levels.

**Implications of all the available evidence**

The findings of our study, along with the findings of the majority of other large population-level studies, suggest that COVID-19 PHSM appear to be linked to increased early childhood developmental concerns and other indicators of poorer developmental outcomes. Our findings highlight the need to target additional support towards children impacted by COVID-19 PHSM, and to continue to monitor their developmental outcomes over time as they get older and more data becomes available. In addition, future pandemic planning should consider possible impacts of PHSM on child development.

## Introduction

The COVID-19 pandemic led to countries around the world implementing Public Health and Social Measures (PHSM) to curb the spread of infections. In Scotland, PHSM in 2020 and 2021 included ‘stay at home’ orders, closures of public places (including schools, nurseries, playgrounds and early learning and childcare settings for most children), travel restrictions, and restrictions on meeting with other households. PHSM also affected care pathways and support for mothers and babies, with many postnatal support and follow up visits being moved to phone calls, online platforms or being postponed. For a full timeline of key events and PHSM policy developments during the pandemic in Scotland, see Table 1.

**Table 1:** Timeline of key events and developments relating to PHSM during the COVID-19 pandemic in Scotland.

Date	Policy developments
11th March 2020	- World Health Organisation declares COVID-19 a pandemic - First case of community transmission of COVID-19 in Scotland
16th March 2020	- Cancellation of mass events of 500 people or more - People advised against unnecessary social contact
20th March 2020	- All schools and nurseries ordered to close - All pubs, restaurants, gyms and other social venues ordered to close
23rd March 2020	- Strict national lockdown announced - People told to only leave their homes to buy food, to exercise for up to 1 h per day or to go to work if they cannot work from home (with fines for non-compliance)
27th March 2020	- Rules on ‘social distancing’ are published
28th April 2020	- New guidance on wearing facemasks in enclosed public spaces introduced
29th May 2020	- Phase 1 of route map out of lockdown begins - Meeting up with one other household outdoors is allowed
3rd June 2020	- Childminder services (limited to children from up to four households) and fully outdoor childcare providers are allowed to reopen
19th June 2020	- Phase 2 of route map out of lockdown begins - Meeting up to eight people from two households is allowed outdoors - Certain households can meet other households indoors as part of ‘extended household’ - Playgrounds can reopen
10th July 2020	- Phase 3 of route map out of lockdown begins in most area - Meeting up to eight people from three households is allowed indoors - Meeting up to fifteen people from five different households is allowed outdoors
13th July 2020	- Organised outdoor play and contact sports for children can resume
15th July 2020	- Indoor restaurants, cafes, pubs, museums, galleries, cinemas, places of worship and libraries can re-open with strict social distancing rules
11th August 2020	- Schools re-open
23rd September 2020	- New restrictions on household visits and a curfew for pubs, bars and restaurants is introduced
5th October 2020	- Restrictions on parent and baby groups relaxed to allow up to ten adults to attend groups where babies are aged <12 months
8th December 2020	- COVID-19 vaccination programme begins
5th January 2021	- New strict ‘level 5’ national lockdown begins - People can only leave their home for essential purposes
20th February 2021	- Children in early learning and childcare, and primaries 1 to 3 return full-time to classrooms
12th March 2021	- Lockdown restrictions ease in certain ‘’level 4′ areas to allow up to four adults from two households to meet up outdoors
16th April 2021	- Restrictions further eased to allow outdoor meetings of up to six adults from six households - Restrictions on travelling outside of local areas are lifted
26th April 2021	- Restrictions eased further to ‘level 3’ throughout Scotland - Restaurants, cafes, pubs, museums, galleries, and libraries can re-open in all areas - Non-essential informal childcare resumes
17th May 2021	- Restrictions eased again to ‘level 2’ in most areas - Indoor gatherings now allowed for up to six people from three households - Social distancing rules relaxed for private gatherings
5th June 2021	- Some areas move to ‘level 1’ restrictions, allowing larger gatherings to take place
19th July 2021	- All areas move to ‘level 0’ restrictions - Larger gatherings allowed and social distancing rules relaxed in public indoor settings
9th August 2021	- All remaining restrictions on gatherings and social distancing are dropped
14th December 2021	- New guidance on reducing social interaction is published due to rising cases of Omicron variant of COVID-19
21st December 2021	- Social distancing rules return in indoor public places and attendances at large events are limited
24th January 2022	- Social distancing rules and limits on attendances at large events are removed
5th May 2023	- World Health Organisation determine that COVID-19 no longer constitutes a public health emergency of international concern

Notes: all policy developments listed applied to Scotland, with some being UK-wide measures and some being Scotland-specific measures. Main information source: Scottish Parliament Information Centre.^1^

Whilst PHSM helped reduce COVID-19 transmission,^2^ they also had potential implications for children going through key developmental periods during the pandemic. Early childhood development is determined by a range of environmental and social factors, as well as biological factors. Importantly, early child developmental outcomes can be influenced greatly by factors such as their caregiver's capacity to provide responsive caregiving, their opportunities for early learning and playing activities, and parental mental health.^3^ During the pandemic, PHSM significantly changed the circumstances of children's lives, by: (1) reducing interaction with others, (2) reducing attendance in early childhood education and childcare settings, (3) potentially exacerbating parental mental health and reducing families' capacity to provide nurturing care due to pandemic impacts on finances, work, relationships and stress. This has potential developmental implications since interaction with others can improve cognitive, language, and socioemotional development and executive functioning,^4^^,^^5^ and parental mental health and nurturing care are key to early childhood development.^3^ Whilst the potential impacts of PHSM on child development are mainly negative, there may also have been some positive implications. For example, PHSM may have reduced prenatal exposure to non-SARS-CoV-2 infections which may be linked to developmental concerns and vulnerabilities.^6^ This may have outweighed any additional risk associated with prenatal exposure to COVID-19. Additionally, some children may have benefitted from more time spent with their immediate family, or by getting involved in different creative activities at home,^7^ although this would only apply to families who were able to provide such opportunities.

In Scotland, existing research, though limited, suggests the pandemic may have been associated with poorer developmental outcomes. For example, descriptive analysis by Public Health Scotland suggests rates of developmental concerns increased during the pandemic.^8^ This is supported by one study analysing linked administrative health data, although this research primarily focussed on the interactive effects of maternal mental health and birth during the pandemic on children's developmental outcomes, rather than the isolated effect of PHSM.^9^ Mixed methods research by Public Health Scotland^10^ has also reported that PHSM had some perceived benefits, such as helping parents maintain good relationships with children. However, this research also identified negative impacts including: (1) severely reduced mixing with others, (2) increased parental stress, (3) difficulties accessing children's services, and (4) poorer child mental health, wellbeing, and developmental outcomes. Surveys in England and Wales have also reported a decline in teacher-reported school readiness among children born or raised during the pandemic.^11^

Internationally, a range of quantitative studies have examined associations between the pandemic, PHSM, and child developmental outcomes. Of these, one reported improved outcomes,^12^ others reported no significant overall changes,^13^^,^^14^ and some reported mixed findings.^15^^,^^16^ Most studies, however, including most large or population-level studies, have highlighted negative developmental outcomes. For example, population-level data from South Korea's National Health Screening Program found associations between the pandemic and delays in communication, cognitive development, social interaction, self-care and fine motor skills among young children.^17^^,^^18^ Large cohort studies in the United States have similarly linked pandemic-related disruption with poorer outcomes in communication, problem solving, and socioemotional development.^19^^,^^20^ Other comparable studies have reported developmental losses and delayed language development among children in Uruguay^21^ and Japan.^22^

Despite this international evidence, there are currently no known large population-level studies specifically examining associations between PHSM and child developmental outcomes in Europe, and review articles have highlighted the lack of extensive longitudinal data.^23^^,^^24^ As pandemic responses varied internationally, data from diverse national contexts are essential for building a comprehensive picture. In the United Kingdom, existing studies are limited by having non-representative, self-selected samples,^10^ or by conducting descriptive analysis only.^8^ Our study, conducted as part of the wider COVID-19 Health Impact on Long-term Child Development in Scotland (CHILDS) study, addressed this gap by using population-level administrative health data to analyse associations between PHSM and early childhood developmental concerns in Scotland.

## Methods

### Study design and participants

We conducted interrupted time series analysis using data from child health reviews among children aged 13–15 months and 27–30 months in Scotland. These are routine reviews of children's health, development and broader wellbeing. During the reviews, information is collected on children's growth (height and weight), development (social, behavioural, communication, motor) and diagnoses, and advice and support is provided to their caregivers.^8^ Reviews are offered to all children in Scotland, and are carried out by trained health visitors, i.e., healthcare professionals who are registered nurses or midwives and have completed additional training to provide specialised support to families.

We included all children who received reviews between January 2019 and August 2023, and had complete developmental data. This included review records for 186,265 children aged 13–15 months and 186,766 children aged 27–30 months, covering 257,532 individual children. This represents approximately 80% of children eligible for each review during the analysis period. This is because 88–90% of eligible children receive each review^25^ and, according to our data, 200,531 children had a 13–15 month review recorded during the analysis period, of whom 186,265 had complete developmental data. Similarly, 208,148 children had a 27–30 month review recorded, with 186,766 having complete developmental data. The remaining 20% of children not included in our analysis for each review is made up of those whose parent or caregiver declined the review, which is not compulsory, or who participated in a review without complete developmental assessment.

### Procedures

In order to obtain descriptive statistics on children taking part in child health reviews, we linked child health review data to data on child sex, child age, child ethnicity, urban-rural classification, Scottish Index of Multiple Deprivation (SIMD, which is a relative measure of area-based deprivation) quintiles and maternal age from the COVID-19 in Pregnancy in Scotland (COPS) administrative dataset.^26^ Review records were used to create weekly time-series data tracking developmental concerns between January 2019 and August 2023. Primary outcomes were the weekly proportion of children with: (1) any developmental concerns at 13–15 months, and (2) any developmental concerns at 27–30 months. This covered concerns relating to speech-language-communication, problem solving, gross motor, personal-social, emotional-behavioural or fine motor development. Additional measures disaggregated these overall outcomes by developmental domain, capturing the weekly proportion of children with specific 13–15 month and 27–30 month concerns in speech-language-communication, problem solving, gross motor, personal-social, emotional-behavioural, and fine motor development. Health visitors evaluate potential concerns through discussions with parents or caregivers, structured observation of the child and the Ages and Stages Questionnaires 3rd Edition (ASQ-3).^27^ Health reviews are typically conducted in the child's home, but during the pandemic many were conducted remotely.^8^

Key dates in PHSM policy developments were used to specify two intervention points in the weekly time-series data. Firstly, the 12th week of 2020 was used as an intervention point indicating the beginning of PHSM, reflecting the initial lockdown being introduced on 23rd March 2020. Secondly, the 32nd week of 2021 was used as an intervention point indicating the ending of PHSM, reflecting the removal of all final remaining social distancing measures on 9th August 2021 (with the exception of the brief re-introduction of some measures between December 2021 and January 2022).

### Statistical analysis

Statistical analysis evaluated associations between the March 2020 introduction of PHSM, their August 2021 removal, and developmental concerns, using interrupted time-series analysis.^28^ Weekly time-series were constructed for each outcome by aggregating individual-level child health review data to give the weekly proportion of children with any developmental concerns, and domain specific concerns. 13–15 month reviews and 27–30 month reviews were assessed separately to allow for the possibility of different effects of PHSM at different developmental stages. Proportions reflect the percentage of children who received a health review each week who were recorded as having developmental concerns.

Autoregressive integrated moving average (ARIMA) models were used to assess associations while adjusting for autocorrelation and pre-existing trends. Models tested for significant ‘slope changes’, i.e., gradual changes in the trend following the March 2020 introduction, and August 2021 removal, of PHSM. Slope changes were examined via assessing the interaction between dummy variables representing the introduction and removal of PHSM and a weekly time term. We evaluated ‘slope changes’ rather than ‘step changes’ (which look at immediate changes) because we hypothesised that any PHSM impacts on child development would likely have developed slowly over time in a ‘ramp’ like effect, rather than there being an immediate impact. This is because when PHSM were introduced it would take time for the effects of reduced social interaction and opportunities for early learning to gradually translate into developmental concerns.

Candidate ARIMA models were selected by testing for autocorrelation via autocorrelation and partial autocorrelation plots. Best fitting models were then chosen based on Akaike information criteria and Bayesian information criteria, with model assumptions (i.e., normality of residuals and absence of autocorrelation) assessed using kernel density plots and portmanteau tests. The first and last week of each calendar year were dropped from the time-series due to limited review activity during public holidays. Similarly, the first three weeks of lockdown in March–April 2020 were also dropped from the time-series due to limited review activity while health services were adapting to new restrictions. These time-series gaps were handled using Kalman filtering methods, whereby state-updating equations are continued without any contributions from the missing data and prediction error for the missing observations are effectively assumed to be zero.^29^ Other than these specific weeks, there were no other gaps in our time-series, and child health reviews were conducted fairly consistently, including during the pandemic period (after the initial few weeks of disruption). See the supplementary appendix Figure S1 (p2) for details of the weekly number of child health reviews conducted during each stage of our analysis period.

In addition to the main analysis, we also conducted four sensitivity analyses. First, we addressed a potential issue related to the implementation of 13–15 month reviews in Greater Glasgow and Clyde, where uptake was low prior to August 2019 due to later review implementation here. We therefore ran a sensitivity analysis restricting the analysis period to post August 2019 for models on 13–15 month outcomes. Secondly, another potential issue relates to the recording of problem solving developmental concerns at 27–30 month reviews in Greater Glasgow and Clyde. Many children here had incomplete problem solving developmental data prior to May 2019. We therefore conducted another sensitivity analysis restricting the analysis period to post May 2019 for 27–30 month outcomes relating to any or problems solving concerns. Thirdly, in order to further test whether these issues relating to health review implementation and recording in Greater Glasgow and Clyde influenced our results, we also conducted additional sensitivity analysis in which we repeated the main analysis but with children from Greater Glasgow and Clyde excluded. Fourthly, a final issue with our main analysis was an unexplained breakpoint in the time-series, whereby the proportion of children with 13–15 month developmental concerns began to rise in 2023. To account for this, we conducted sensitivity analysis in which the analysis period was narrowed to January 2019 to December 2022 for 13–15 month concerns.

All analyses were conducted within Scotland's National Safe Haven using Stata IC version 16.1. Our analysis plan was preregistered using Open Science Framework (https://osf.io/r598z). All analyses were included in this plan, except for the third and fourth pieces of sensitivity analysis outlined above, which were unplanned and added following peer reviewer comments and post-analysis visual inspection of time series graphs. Results are reported in accordance with the RECORD guidelines (see supplementary appendix Table S1, p3-7, for RECORD checklist).

### Ethics approval

Ethical approval was granted by the School of Philosophy, Psychology and Language Sciences Research Ethics Committee at the University of Edinburgh (reference 15–2223/2). Information governance approval was provided by the NHS Scotland Public Benefit and Privacy Panel for Health and Social Care (Reference 2021–0178).

### Role of the Funding source

Funders played no role in our study design, data collection, analysis, interpretation, or writing of the report.

## Results

Table 2 presents descriptive statistics on key sociodemographic characteristics of participants across each stage of the analysis period. The 186,265 records from 13 to 15 month health reviews included 95,506 male (51.3%) and 90,756 female children (48.7%). Among these, 167,694 (90.0%) children were from White ethnic groups, 8131 (4.4%) from Asian ethnic groups, 5296 (2.8%) from Mixed ethnic groups, 3088 (1.7%) from Black ethnic groups and 120 (0.1%) from unknown ethnic groups, where ethnicity data was missing. The 186,766 records from 27 to 30 month health reviews included 95,209 (51.0%) male and 91,557 (49.0%) female children, 169,739 (90.9%) children from White ethnic groups, 7640 (4.1%) from Asian ethnic groups, 4862 (2.6%) from Mixed ethnic groups, 2776 (1.5%) from Black ethnic groups and 94 (<0.1%) from unknown ethnic groups. Notably, children from White ethnic groups are slightly over-represented in our study, as analysis from Public Health Scotland suggests that child health review coverage is higher for White ethnic groups.^30^ At 13–15 months, for example, they make up 90% of our participants despite only making up about 88% of eligible children.^30^ Additional descriptive statistics on developmental concerns in each part of the analysis period are provided in the supplementary appendix (Table S2, p8-9).

**Table 2:** Characteristics of study population in each part of analysis period, 13–15 month child health reviews and 27–30 month child health reviews.

	13–15 Month Child Health Reviews				27–30 Month Child Health Reviews			
	Total Analysis period	Pre PHSM Period	During PHSM Period	Post PHSM Period	Total Analysis period	Pre PHSM Period	During PHSM Period	Post PHSM Period
**Total**	186,265 (100%)	49,131 (100%)	54,898 (100%)	82,236 (100%)	186,766 (100%)	50,461 (100%)	55,889 (100%)	80,416 (100%)
**Child sex**								
Male	95,506 (51.3%)	25,263 (51.4%)	28,067 (51.1%)	42,176 (51.3%)	95,209 (51.0%)	25,740 (51.0%)	28,637 (51.2%)	40,832 (50.8%)
Female	90,756 (48.7%)	23,868 (48.6%)	26,831 (48.9%)	40,060 (48.7%)	91,557 (49.0%)	24,721 (49.0%)	27,252 (48.8%)	39,584 (49.2%)
**Mean child age, months (SD)**	14.30 (1.40)	14.20 (1.45)	14.26 (1.48)	14.39 (1.39)	29.00 (1.90)	28.75 (1.73)	29.03 (2.02)	29.12 (1.90)
**Child ethnicity**								
White ethnic group	167,694 (90.0%)	44,773 (91.1%)	49,610 (90.4%)	73,311 (89.1%)	169,739 (90.9%)	46,453 (92.1%)	50,781 (90.9%)	72,505 (90.2%)
Black ethic group	3088 (1,7%)	696 (1.4%)	856 (1.6%)	1536 (1.9%)	2776 (1.5%)	664 (1.3%)	814 (1.5%)	1298 (1.6%)
Asian ethnic group	8131 (4.4%)	1855 (3.8%)	2376 (4.3%)	3900 (4.7%)	7640 (4.1%)	1805 (3.6%)	2260 (4.0%)	3575 (4.4%)
Mixed ethnic group	5296 (2.8%)	1353 (2.8%)	1484 (2.7%)	2459 (3.0%)	4862 (2.6%)	1152 (2.3%)	1522 (2.7%)	2188 (2.7%)
Other ethnic group	1936 (1.0%)	433 (0.9%)	548 (1.0%)	955 (1.2%)	1655 (0.9%)	364 (0.7%)	489 (0.9%)	802 (1.0%)
Unknown	120 (0.1%)	21 (<0.1%)	24 (<0.1%)	75 (0.1%)	94 (<0.1%)	23 (<0.1)	23 (<0.1%)	48 (0.1%)
**Urban-rural classification**								
Urban	156,633 (84.1%)	41,462 (84.4%)	46,176 (84.1%)	68,995 (83.9%)	157,937 (84.6%)	42,764 (84.8%)	47,474 (84.9%)	67,699 (84.2%)
Rural	29,632 (15.9%)	7669 (15.6%)	8722 (15.9%)	13,241 (16.1%)	28,829 (15.4%)	7697 (15.2%)	8415 (15.1%)	12,717 (15.8%)
**SIMD quintile**								
1 (most deprived)	43,561 (23.4%)	11,249 (22.0%)	13,220 (24.1%)	19,092 (23.2%)	44,324 (23.7%)	11,770 (23.3%)	21,766 (23.5%)	19,063 (23.7%)
2 (more deprived)	38,373 (20.6%)	10,393 (21.2%)	11,436 (20.8%)	16,544 (20.1%)	38,596 (20.7%)	10,513 (20.8%)	19,330 (20.9%)	16,433 (20.4%)
3 (medium deprived)	34,266 (18.4%)	9142 (18.6%)	10,087 (18.4%)	15,037 (18.3%)	34,253 (18.3%)	9469 (18.8%)	17,113 (18.5%)	14,680 (18.3%)
4 (less deprived)	38,402 (20.6%)	10,020 (20.4%)	11,109 (20.2%)	17,273 (21.0%)	37,770 (20.2%)	10,106 (20.0%)	18,810 (20.4%)	16,510 (20.5%)
5 (least deprived)	31,663 (17.0%)	8327 (16.9%)	9046 (16.5%)	14,290 (17.4%)	31,823 (17.0%)	8603 (17.1%)	15,401 (16.7%)	13,729 (17.1%)
**Maternal age-band at child's birth**								
<20 years	4889 (2.6%)	1545 (3.1%)	1531 (2.8%)	1813 (2.2%)	5630 (3.0%)	1751 (3.5%)	1728 (3.1%)	2151 (2.7%)
20–35 years	148,075 (79.5%)	39,152 (79.7%)	43,705 (79.6%)	65,218 (79.3%)	148,699 (79.6%)	40,378 (80.0%)	44,467 (79.6%)	63,854 (79.4%)
>35 years	33,301 (17.9%)	8434 (17.2%)	9662 (17.6%)	15,205 (18.5%)	32,437 (17.4%)	8332 (16.5%)	9694 (17.3%)	14,411 (17.9%)

Notes: Data are N (%) unless otherwise indicated. The total analysis period was January 2019–August 2023, the pre PHSM period was January 2019–March 2020, the during PHSM period was March 2020–August 2021 and the post PHSM period was August 2021–August 2023.

**Fig. 1:**
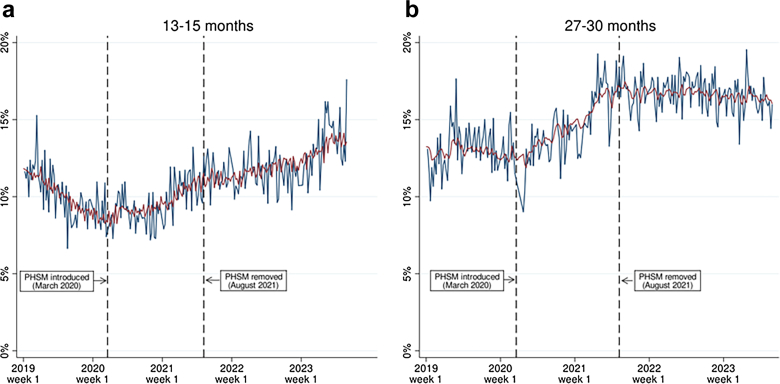
Weekly proportion of children with any (i.e., at least one) developmental concern(s) at 13–15 month and 27–30 month health reviews, raw time-series vs. model values. Notes: the raw time series is shown in blue and ARIMA model values are shown in red.

**Fig. 2:**
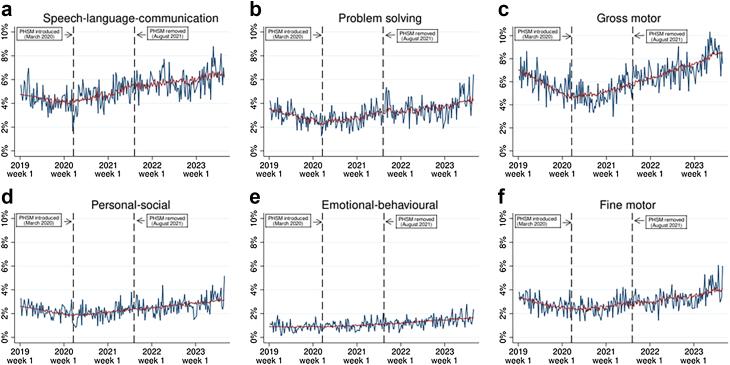
Weekly proportion of children with domain-specific developmental concerns at 13–15 month health reviews, raw time-series vs. model values. Notes: the raw time series is shown in blue and ARIMA model values are shown in red.

**Fig. 3:**
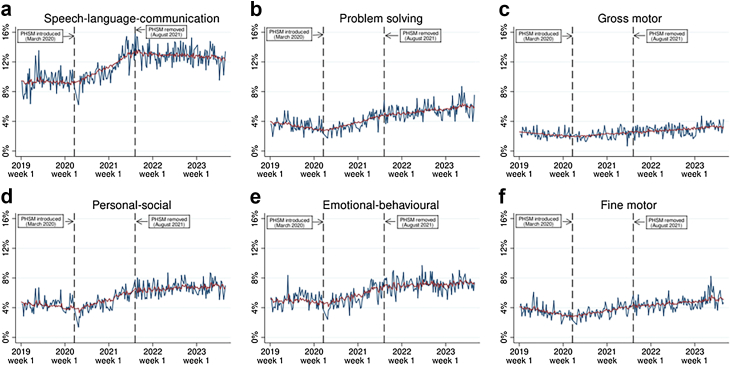
Weekly proportion of children with domain-specific developmental concerns at 27–30 month health reviews, raw time-series vs. model values. Notes: the raw time series is shown in blue and ARIMA model values are shown in red.

Results of the interrupted time series analysis are summarised in Table 3, with model values also shown alongside the raw time series for each outcome in Fig. 1, Fig. 2, Fig. 3. The introduction of PHSM in March 2020 was associated with a slope change increase in the proportion of children with any developmental concerns (+0.091 percentage points per week [95% CI: 0.065, 0.116] at 13–15 months and +0.076 percentage points per week [95% CI: 0.048, 0.104] at 27–30 months). To put these figures into context, +0.091 percentage points per week corresponds to around a 4.7 percentage points increase per year, or a 6.6 percentage points increase overall across the full 72 weeks of PHSM being in place from March 2020–August 2021. Meanwhile, +0.076 percentage points per week corresponds to around a 4.0 percentage points increase per year or 5.5 percentage points increase overall across the 72 weeks of PHSM. The August 2021 PHSM removal was associated with a slope change decrease in the proportion of children with any developmental concerns at 27–30 months (−0.067 percentage points per week [95% CI: −0.088, −0.046]) but was not significantly associated with any 13–15 month slope changes (−0.010 percentage points per week [95% CI: −0.025, 0.006]). This is illustrated in Fig. 1. Panel (a) shows that the proportion of children with any 13–15 month developmental concerns increased following PHSM introduction and continued to rise gradually even after PHSM were lifted. Panel (b) shows that the proportion of children with any 27–30 month developmental concerns increased after PHSM were introduced before levelling off after PHSM were removed, although it remained higher than pre-pandemic levels.

**Table 3:** Associations between COVID-19 PHSM introduction, COVID-19 PHSM removal and slope changes in the weekly proportion of children with developmental concerns identified at 13–15 month and 27–30 month child health reviews.

	13–15 month child health reviews, slope change B (95% CI)				27–30 month child health reviews, slope change B (95% CI)			
	PHSM introduction	PHSM removal	Constant	AR terms	PHSM introduction	PHSM removal	Constant	AR terms
Any developmental concerns	0.091 (0.065, 0.116)	−0.010 (−0.025, 0.006)	185.527 (132.843, 238.211)	L3: 0.200 (0.062, 0.336) L36: −0.243 (−0.396, −0.090)	0.076 (0.048, 0.104)	−0.067 (−0.088, −0.046)	55.593 (−1.351, 112.537)	L1: 0.166 (0.034, 0.299) L2: 0.162 (0.026, 0.298)
Speech-language-communication concerns	0.033 (0.019, 0.046)	−0.012 (−0.021, −0.003)	42.904 (14.374, 71.434)	L29: −0.247 (−0.399, −0.095) L37: −0.077 (−0.223, 0.074)	0.063 (0.041, 0.086)	−0.064 (−0.079, −0.048)	27.924 (−16.053, 71.901)	L2: 0.166 (0.010, 0.322)
Problem solving concerns	0.031 (0.015, 0.046)	−0.003 (−0.011, 0.006)	62.401 (28.763, 96.039)	L1: 0.160 (0.031, 0.289) L35: −0.176 (−0.341, −0.012)	0.049 (0.031, 0.066)	−0.016 (−0.027, −0.006)	66.072 (29.656, 102.489)	L1: 0.167 (0.003, 0.331)
Gross motor concerns	0.056 (0.039, 0.072)	0.010 (−0.001, 0.022)	129.801 (94.544, 165.058)	L3: 0.157 (0.004, 0.309)	0.018 (0.008, 0.028)	−0.001 (−0.007, 0.005)	34.411 (11.867, 56.955)	L25: −0.179 (−0.316, −0.042)
Personal-social concerns	0.020 (0.011, 0.029)	<0.001 (−0.005, 0.005)	41.991 (22.118, 61.865)	L29: −0.144 (−0.309, 0.020)	0.052 (0.035, 0.070)	−0.028 (−0.040, −0.017)	56.676 (18.526, 94.827)	L1: 0.256 (0.113, 0.923)
Emotional-behavioural concerns	0.003 (−0.003, 0.010)	0.002 (−0.002, 0.005)	1.746 (−10.605, 14.096)	L20: −0.117 (−0.267, 0.036)	0.042 (0.020, 0.064)	−0.025 (−0.040, −0.011)	37.628 (−9.603, 84.860)	L1: 26.77 (0.142, 0.393)
Fine motor concerns	0.023 (0.011, 0.036)	0.003 (−0.004, 0.011)	53.967 (27.109, 80.825)	L4: 0.182 (0.033, 0.331) L20: −0.163 (−0.319, −0.006)	0.039 (0.022, 0.057)	−0.009 (−0.019, 0.002)	68.608 (33.567, 103.649)	L1: 0.193 (0.059, 0.328)

ARIMA model estimates adjust for autocorrelation and trend. PHSM introduction was the 12th week of 2020, reflecting the fact that strict lockdown measures were first introduced in Scotland on 23rd March 2020. PHSM removal was the 32nd week of 2021, reflecting the fact that all remaining social distancing measures were removed on 9th August 2021 (this is used as an indicator of the general ending of PHSM, although some restrictions were temporarily reintroduced in December–January 2021). B = coefficient, 95% CI = 95% confidence intervals. Results for the AR terms included in each model are provided next to main results, with L referring to the number of lags. These were selected following an iterative process involving autocorrelation function and partial autocorrelation function plots and model fit statistics.

For domain specific developmental concerns, the March 2020 PHSM introduction was associated with a significant slope change increase in the proportion of children with concerns across all domains at both 13–15 months and 27–30 months, with the exception of emotional and behavioural concerns at 13–15 months, which showed no significant slope change. The August 2021 removal of PHSM was not associated with any significant slope changes in the proportion of children with developmental concerns in any domains at 13–15 months. However, it was associated with significant a slope change decrease in the proportion of children with developmental concerns at 27–30 months in all domains except problem solving and fine motor development. These findings are illustrated in Figs. 2 and 3. Fig. 2 highlights how the proportion of children with domain-specific developmental concerns at 13–15 months generally began to gradually rise after PHSM were introduced in March 2020, and continued to rise (even if at a slower pace) after PHSM were removed in August 2021. Fig. 3, on the other hand, shows that the proportion of children with domain-specific developmental concerns at 27–30 months began to gradually rise after PHSM were introduced, but then (except for gross motor and fine motor concerns) these rises slowed substantially or stopped completely after PHSM were removed.

Kernel density plots and portmanteau statistics assessing model assumptions (i.e., normality of residuals and absence of autocorrelation) are available in the supplementary appendix (Model assumption checks, p10-23). Sensitivity analysis results are also provided in the supplementary appendix (Tables S3–S6, p24-29). The sensitivity analysis restricting the analysis period for 13–15 month outcomes to post August 2019 (due to lower review coverage in Greater Glasgow and Clyde prior to that date) showed the results were consistent with the main analysis for the primary outcome (any concerns). However, slope change increases for domain specific concerns related to speech-language-communication, problem solving, personal-social development and fine-motor skills were no longer statistically significant. The results of the sensitivity analysis with the narrower, i.e., post May 2019, analysis period for 27–30 month outcomes relating to any or problem solving concerns (due to incomplete data on problem solving concerns in Greater Glasgow and Clyde prior to this) were consistent with the main analysis. The sensitivity analysis excluding children from Greater Glasgow and Clyde (again due to the later implementation and recording of problem solving concerns there) was largely consistent with the main analysis, except that, at 13–15 months, PHSM removal was now associated with significant slope change decreases in any developmental concerns (−0.031 percentage points per week [95% CI: −0.052, −0.011]) and domain specific concerns relating to problem solving and fine motor concerns. Finally, in the sensitivity analysis with the analysis period restricted to January 2019–December 2022 for 13–15 month concerns (to account for the unexplained time series breakpoint from January 2023), the August 2021 PHSM removal was now associated with slope change decreases in any concerns (−0.031 percentage points per week [95% CI: −0.052, −0.011]) as well as domain-specific concerns related to speech-language-communication, problem solving, personal-social and fine motor development.

## Discussion

Our study, conducted as part of the wider CHILDS study, utilised child health review records for children aged 13–15 months and 27–30 months in Scotland to examine associations between COVID-19 PHSM and child developmental concerns. Our findings suggest the March 2020 introduction of PHSM was associated with significant slope change increases in the proportion of children with developmental concerns at both 13–15 month and 27–30 month health reviews. Our findings of a +0.091 percentage points per week slope change increase in the proportion of children with any 13–15 month developmental concerns and a +0.076 percentage points per week slope change increase in the proportion of children with any 27–30 month developmental concerns, correspond to an estimated +5.5–6.6 percentage points increase overall when considered in the context of the full 72 weeks of PHSM from March 2020–August 2021. Following on from this, our main analysis findings suggest that the proportion of children with 13–15 month concerns continued to rise even after PHSM were removed in August 2021, although sensitivity analysis suggested that after accounting for the data breakpoint in 2023 the proportion of children with concerns stopped increasing following PHSM removal. Meanwhile, the proportion of children with 27–30 month developmental concerns stopped increasing once PHSM ended, although levels remained higher than pre-pandemic. These results were broadly consistent across developmental domains.

Our findings are consistent with previous descriptive analysis of Scottish child development data,^8^ and international evidence from population-level analyses and large cohort studies from South Korea, the USA, Uruguay and Japan.^17^, ^18^, ^19^, ^20^, ^21^, ^22^ To our knowledge, our study is the first to specifically model associations between COVID-19 PHSM and child developmental outcomes using population-level statistics from the United Kingdom or Europe. Our findings highlight the need to target additional support towards children impacted by COVID-19 PHSM, and to continue to monitor their developmental outcomes over time as they get older and more data becomes available. In addition, any future pandemic planning should take into account the possible impacts that implementing PHSM may have on childhood development, and consider targeted interventions to mitigate any adverse impacts on children. This may include efforts to prioritise keeping children's services, parks and playgrounds operating where possible, and to support caregivers in ensuring children continue to have early learning opportunities.

However, our study has some limitations. Importantly, it is difficult to disentangle PHSM effects from other concurrent changes. One potential issue is that that the pandemic may have led to a self-fulfilling rise in concerns, e.g., if health visitors (or parents or caregivers who were informing reviews) were worried about potential impacts of children's lack of socialising on their development then this could have increased their propensity to classify issues as developmental concerns. This type of interrupted time series analysis also does not control for any secular trends over the analysis period in underlying sociodemographic characteristics of children. Moreover, PHSM led to many health reviews temporarily shifting from in-person to phone calls or video conferencing.^8^ We do not have data on which reviews were conducted in-person or remotely. April 2020 guidance stated reviews should be conducted remotely if possible, before May 2020 guidance allowed resumption of in-person reviews if particular conditions were met. Consequently, most reviews were conducted remotely during the initial lockdown before a gradual return to in-person (but this likely varied by area and family needs). This could bias our analysis if delivery mode changes altered the likelihood of developmental concerns being identified. However, if this were the case, one might expect to see an immediate change in the proportion of concerns following the April 2020 guidance to shift to remote reviews, rather than the gradual increase observed. This suggests that changes are more likely due to genuine PHSM impacts, although the fact that concerns did not decline to pre-pandemic levels after PHSM ended indicates that other factors may also have contributed (or that PHSM effects were lagged).

Another limitation relates to pandemic-related disruption to review delivery. Notably, the weekly number of health reviews being conducted fell sharply for a few weeks following the initial March 2020 lockdown (which is why we treated these weeks as missing data) before returning to pre-pandemic levels by late April 2020. This disruption could have biased our analysis if review delays or subsequent backlog affected the likelihood of concerns being detected. For instance, some developmental difficulties can be more readily identified in older children, thus increasing likelihood of concerns being detected (since delays may have led to children being slightly older when reviewed). Conversely, concerns that self-resolve with time (e.g., slightly late walking) would not be picked up in older children. Table 2 indicates that children reviewed during the pandemic period were slightly older, although differences were relatively small. It should also be noted that health visitors would be using the ASQ version appropriate to the child's age.

A further limitation is variation in the implementation of reviews and data completeness across health boards. Greater Glasgow and Clyde had: (1) low coverage of 13–15 month health reviews prior to August 2019, and (2) high levels of incomplete data on 27–30 month problem solving concerns prior to May 2019. As our analysis period began in January 2019, these issues may have influenced our results. In our sensitivity analysis, restricting the analysis period to post-August 2019 for 13–15 month concerns did not significantly alter the primary outcome (any concerns), although associations for some domain-specific concerns became non-significant, suggesting a possible influence of this issue. Restricting the 27–30 month analysis to post-May 2019 for any and problem solving concerns did not materially change our results. Results of the sensitivity analysis excluding children from Greater Glasgow and Clyde were also broadly in line with the main analysis. One final consideration regarding data completeness is that the pandemic may have impacted review coverage in a way that biases our results if it led to more selective delivery or uptake of reviews depending on perception of needs. Reassuringly, analysis by Public Health Scotland^30^ suggests that child health review coverage has generally been consistent across key characteristics like child sex and socioeconomic deprivation, and this did not change during the pandemic. However, as noted above in our results section, children taking part in child health reviews are not completely representative of all children in Scotland with regards to ethnicity, as White ethnic groups are slightly over-represented.^30^ Our findings may also not be generalisable internationally due to differences in PHSM between Scotland and other countries.

To conclude, COVID-19 PHSM substantially altered the everyday environments of young children in Scotland and globally. Our findings suggest PHSM were associated with increases in developmental concerns among children aged 13–15 months and 27–30 months. These increases levelled off after PHSM were removed, but the proportion of children with developmental concerns remained higher than pre-pandemic levels. These findings are consistent with a causal impact of PHSM, however the influence of potential time-varying confounders cannot be ruled out. Future research will assess whether these increases were distributed evenly across social groups, by examining inequalities (including socioeconomic inequalities, urban-rural inequalities, and inequalities across child sex and ethnicity) in rates of developmental concerns before and during the pandemic. Further research will also look at longer-term effects on the pandemic cohort of children, and explore associations between PHSM and developmental concerns in older children, which can help contribute to existing knowledge on the effects of the pandemic on school readiness, chronic absenteeism and the mental health of young people.^1^^,^^31^

## Contributors

**IH**: conceptualisation, data curation, project administration, formal analysis, methodology, writing – original draft. **LM**: conceptualisation, funding acquisition, writing – review and editing. **AM**: conceptualisation, funding acquisition, writing – review and editing. **JK**: conceptualisation, writing – review and editing. **KO**: conceptualisation, data curation – review and verification, formal analysis – review and verification, writing – review and editing. **LF**: conceptualisation, methodology, writing – review and editing. **AKS**: methodology, writing – review and editing. **JPB**: conceptualisation, writing – review and editing. **MVL**: writing – review and editing. **SJS**: funding acquisition, writing – review and editing. **RW**: conceptualisation, funding acquisition, writing: review and editing. **BA**: conceptualisation, methodology, supervision, project administration, funding acquisition, writing – review and editing. **IH** and **KO** accessed and verified the raw data. All other authors could access the raw data if they wished, provided that they completed information governance training in order to be granted access to the data in Scotland’s National Safe Haven by the Electronic Data Research and Innovation Service (eDRIS) team at Public Health Scotland. All authors had final responsibility for the decision to submit for publication.

## Data sharing statement

The administrative health datasets used for this study can be accessed only by successfully applying to the National Health Service Scotland Public Benefit and Privacy Panel for Health and Social Care (HSC-PBPP). The eDRIS team at Public Health Scotland can assist with the process of applying for approval to access the datasets and, if approved, can facilitate access to the datasets within Scotland National Safe Haven. The authors of the present study cannot directly share the datasets with any others outside of the approved team of researchers working on the CHILDS study.

## Declaration of interests

**IH** declares support from the Economic and Social Research Council. **LM** declares support from UK Research and Innovation, Health Data Research UK and the British Science Association, and a board role at Parenting Across Scotland. **AKS** declares support from the National Institute for Health and Care Research School for Public Health Research. **SJS** declares support from Wellcome Clinical Career Development Fellowship, Tommy's Charity, Medical Research Council, National Institute for Health and Care Research Health Technology and Assessment and Efficacy and Mechanism Evaluation programmes, Chief Scientist Office Scotland and Medical Research Council, consultancy fees from Natera and Norgine and board participation or role at National Institute of Healthcare Research Health Technology and Assessment Data Monitoring Committee, Trial Steering Committee and Sands Charity. **RW** declares support from the Economic and Social Research Council. **BA** declares support from European Union's Horizon 2020 research and innovation programme under the Marie Skłodowska-Curie grant agreement No.813546, the Baily Thomas Charitable Fund, the University of Edinburgh Data Driven Innovation Fund and the Economic and Social Research Council. Other authors declared no interests.
