# Supplementary material for: COVID-19 public health and social measures (PHSM) and early childhood developmental concerns in Scotland: an interrupted time series analysis
Source: Lancet Reg Health Eur. 2025 Nov 25;60:101525. doi: 10.1016/j.lanepe.2025.101525 (PMC12769819; doi:10.1016/j.lanepe.2025.101525)
Supplement: Multimedia component 2 [file mmc2.docx]

**Supplementary Appendix**

**Table of Contents**

[**Figure S1. Weekly number of child health reviews conducted during our analysis period** 2](#_Toc207097544)

[**Table S1. The RECORD statement – checklist of items, extended from the STROBE statement, that should be reported in observational studies using routinely collected health data.** 3](#_Toc207097545)

[**Table S2. Developmental concerns in each part of analysis period, 13-15 month child health reviews and 27-30 month child health reviews** 8](#_Toc207097546)

[**Model Assumption Checks** 10](#_Toc207097547)

[**Table S3. Sensitivity analysis (narrower analysis period of August 2019-August 2023) showing associations between COVID-19 PHSM introduction, COVID-19 PHSM removal and slope changes in the weekly proportion of children with developmental concerns identified at 13-15 month child health reviews** 24](#_Toc207097548)

[**Table S4. Sensitivity analysis (narrower analysis period of May 2019-August 2023) showing associations between COVID-19 PHSM introduction, COVID-19 PHSM removal and slope changes in the weekly proportion of children with developmental concerns identified at 27-30 month child health reviews** 25](#_Toc207097549)

[**Table S5. Sensitivity analysis (Greater Glasgow & Clyde health board excluded) showing associations between COVID-19 PHSM introduction, COVID-19 PHSM removal and slope changes in the weekly proportion of children with developmental concerns identified at 13-15 month and 27-30 month child health reviews** 26](#_Toc207097550)

[**Table S6. Sensitivity analysis (narrower analysis period of January 2019-December 2022) showing associations between COVID-19 PHSM introduction, COVID-19 PHSM removal and slope changes in the weekly proportion of children with developmental concerns identified at 13-15 month child health reviews** 28](#_Toc207097551)

# **Figure S1. Weekly number of child health reviews conducted during our analysis period**


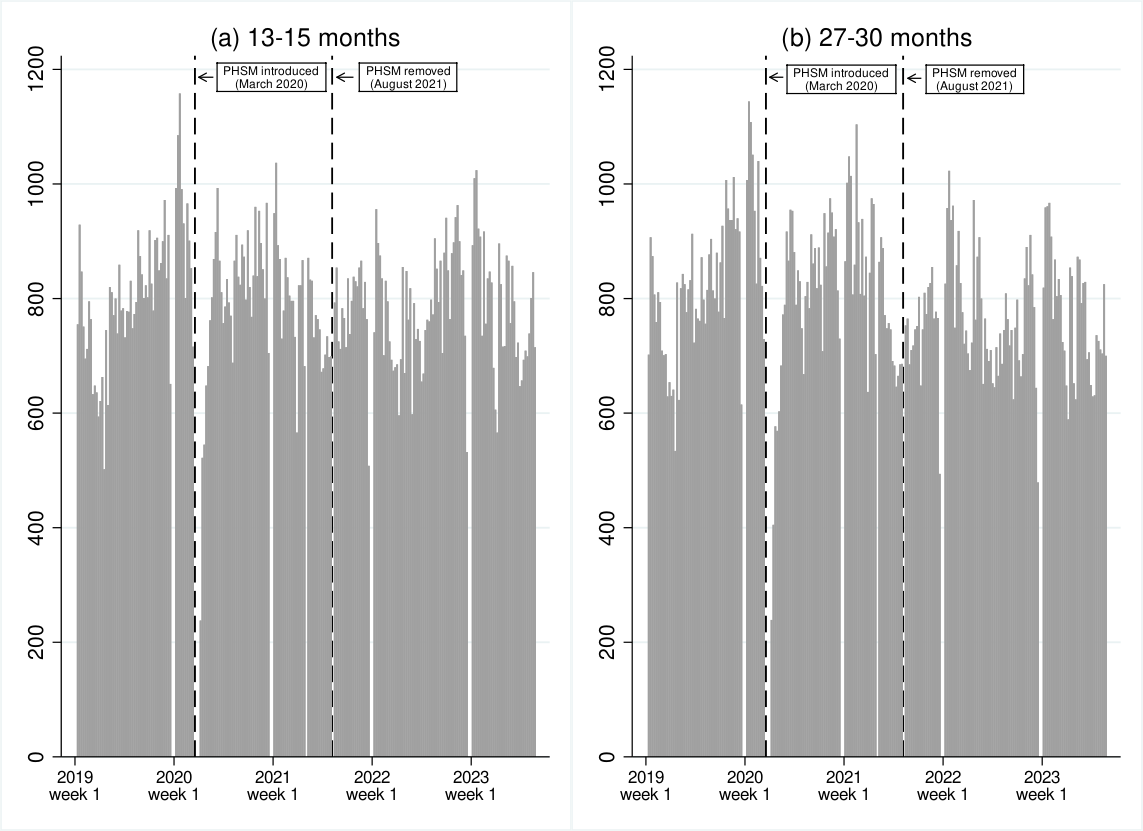


# **Table S1. The RECORD statement – checklist of items, extended from the STROBE statement, that should be reported in observational studies using routinely collected health data.**

|  | **Item No.** | **STROBE items** | **Location in manuscript where items are reported** | **RECORD items** | **Location in manuscript where items are reported** |
| --- | --- | --- | --- | --- | --- |
| **Title and abstract** | | | | | |
|  | 1 | (a) Indicate the study’s design with a commonly used term in the title or the abstract (b) Provide in the abstract an informative and balanced summary of what was done and what was found | 1. (a) title (manuscript p1) and abstract (manuscript p2) | RECORD 1.1: The type of data used should be specified in the title or abstract. When possible, the name of the databases used should be included.  RECORD 1.2: If applicable, the geographic region and timeframe within which the study took place should be reported in the title or abstract.  RECORD 1.3: If linkage between databases was conducted for the study, this should be clearly stated in the title or abstract. | 1.1: Abstract methods section (manuscript p2)  1.2. Abstract methods section (manuscript p2)  1.3. Abstract methods section (manuscript p2) |
| **Introduction** | | | | | |
| Background rationale | 2 | Explain the scientific background and rationale for the investigation being reported | 2. Introduction (manuscript p3-4) |  |  |
| Objectives | 3 | State specific objectives, including any prespecified hypotheses | 3. Introduction (manuscript p4) |  |  |
| **Methods** | | | | | |
| Study Design | 4 | Present key elements of study design early in the paper | 4. Study design and participants part of methods section (manuscript p5) |  |  |
| Setting | 5 | Describe the setting, locations, and relevant dates, including periods of recruitment, exposure, follow-up, and data collection | 5. Study design and participants part of methods section (manuscript p5) |  |  |
| Participants | 6 | *(a) Cohort study* - Give the eligibility criteria, and the sources and methods of selection of participants. Describe methods of follow-up  *Case-control study* - Give the eligibility criteria, and the sources and methods of case ascertainment and control selection. Give the rationale for the choice of cases and controls  *Cross-sectional study* - Give the eligibility criteria, and the sources and methods of selection of participants  *(b) Cohort study* - For matched studies, give matching criteria and number of exposed and unexposed  *Case-control study* - For matched studies, give matching criteria and the number of controls per case | 6. (a) Study design and participants part of methods section (manuscript p5)  6. (b) n/a | RECORD 6.1: The methods of study population selection (such as codes or algorithms used to identify subjects) should be listed in detail. If this is not possible, an explanation should be provided.  RECORD 6.2: Any validation studies of the codes or algorithms used to select the population should be referenced. If validation was conducted for this study and not published elsewhere, detailed methods and results should be provided.  RECORD 6.3: If the study involved linkage of databases, consider use of a flow diagram or other graphical display to demonstrate the data linkage process, including the number of individuals with linked data at each stage. | 6.1. Study design and participants part of methods section (manuscript p5)  6.2. Study design and participants part of methods section (manuscript p5)  6.3. Study design and participants part of methods section – provided in text rather than visually due to limits on number of tables and figures (manuscript p5) |
| Variables | 7 | Clearly define all outcomes, exposures, predictors, potential confounders, and effect modifiers. Give diagnostic criteria, if applicable. | 7. Procedures part of methods section (manuscript p5) | RECORD 7.1: A complete list of codes and algorithms used to classify exposures, outcomes, confounders, and effect modifiers should be provided. If these cannot be reported, an explanation should be provided. | 7.1. Procedures part of methods section (manuscript p5) |
| Data sources/ measurement | 8 | For each variable of interest, give sources of data and details of methods of assessment (measurement).  Describe comparability of assessment methods if there is more than one group | 8. Procedures part of methods section (manuscript p5) |  |  |
| Bias | 9 | Describe any efforts to address potential sources of bias | 9. Statistical analysis part of methods section (manuscript p6) |  |  |
| Study size | 10 | Explain how the study size was arrived at | 10. Study design and participants part of methods section (manuscript p5) |  |  |
| Quantitative variables | 11 | Explain how quantitative variables were handled in the analyses. If applicable, describe which groupings were chosen, and why | 11. Procedures part of methods section (manuscript p5) |  |  |
| Statistical methods | 12 | (a) Describe all statistical methods, including those used to control for confounding  (b) Describe any methods used to examine subgroups and interactions  (c) Explain how missing data were addressed  (d) *Cohort study* - If applicable, explain how loss to follow-up was addressed  *Case-control study* - If applicable, explain how matching of cases and controls was addressed  *Cross-sectional study* - If applicable, describe analytical methods taking account of sampling strategy  (e) Describe any sensitivity analyses | 12. (a) Statistical analysis part of methods section (manuscript p6)  12. (b) Statistical analysis part of methods section (manuscript p6)  12. (c) Study design and participants part of methods section (manuscript p5)  12. (d) Study design and participants part of methods section (manuscript p5)  12. (e) Statistical analysis part of methods section (manuscript p6) |  |  |
| Data access and cleaning methods |  | .. |  | RECORD 12.1: Authors should describe the extent to which the investigators had access to the database population used to create the study population.  RECORD 12.2: Authors should provide information on the data cleaning methods used in the study. | 12.1. Study design and participants part of methods section (manuscript p5) and procedures part of methods section (manuscript p5)  12.2. Procedures part of methods section (manuscript p5) |
| Linkage |  | .. |  | RECORD 12.3: State whether the study included person-level, institutional-level, or other data linkage across two or more databases. The methods of linkage and methods of linkage quality evaluation should be provided. | 12.3. Study design and participants part of methods section (manuscript p5) and procedures part of methods section (manuscript p5) |
| **Results** | | | | | |
| Participants | 13 | (a) Report the numbers of individuals at each stage of the study (*e.g.*, numbers potentially eligible, examined for eligibility, confirmed eligible, included in the study, completing follow-up, and analysed)  (b) Give reasons for non-participation at each stage.  (c) Consider use of a flow diagram | 13. (a) Study design and participants part of methods section (manuscript p5) | RECORD 13.1: Describe in detail the selection of the persons included in the study (*i.e.,* study population selection) including filtering based on data quality, data availability and linkage. The selection of included persons can be described in the text and/or by means of the study flow diagram. | 13.1. Study design and participants part of methods section (manuscript p5) |
| Descriptive data | 14 | (a) Give characteristics of study participants (*e.g.*, demographic, clinical, social) and information on exposures and potential confounders  (b) Indicate the number of participants with missing data for each variable of interest  (c) *Cohort study* - summarise follow-up time (*e.g.*, average and total amount) | 14. (a) Table 2 and results section (p7 of manuscript). Also Table S2 in supplementary appendix (p7-8 of supplementary appendix).  14. (b) Table 2 in results section (p7 of manuscript)  14. (c) Table S2 in supplementary appendix (p7-8 of supplementary appendix). |  |  |
| Outcome data | 15 | *Cohort study* - Report numbers of outcome events or summary measures over time  *Case-control study* - Report numbers in each exposure category, or summary measures of exposure  *Cross-sectional study* - Report numbers of outcome events or summary measures | 15. Table S2 in supplementary appendix (p7-8 of supplementary appendix). |  |  |
| Main results | 16 | (a) Give unadjusted estimates and, if applicable, confounder-adjusted estimates and their precision (e.g., 95% confidence interval). Make clear which confounders were adjusted for and why they were included  (b) Report category boundaries when continuous variables were categorized  (c) If relevant, consider translating estimates of relative risk into absolute risk for a meaningful time period | 16. (a) Table 3 and results section (p7 of manuscript).  16. (b) n/a  16. (c) Discussion section (p8 of manuscript) |  |  |
| Other analyses | 17 | Report other analyses done—e.g., analyses of subgroups and interactions, and sensitivity analyses | 17. Tables S3-S5 in supplementary appendix (supplementary appendix p23-26), and results section (p8 of manuscript) |  |  |
| **Discussion** | | | | | |
| Key results | 18 | Summarise key results with reference to study objectives | 18. Discussion section (p8 of manuscript) |  |  |
| Limitations | 19 | Discuss limitations of the study, taking into account sources of potential bias or imprecision. Discuss both direction and magnitude of any potential bias | 19. Discussion section (p9 of manuscript) | RECORD 19.1: Discuss the implications of using data that were not created or collected to answer the specific research question(s). Include discussion of misclassification bias, unmeasured confounding, missing data, and changing eligibility over time, as they pertain to the study being reported. | 19.1. Discussion section (p9 of manuscript) |
| Interpretation | 20 | Give a cautious overall interpretation of results considering objectives, limitations, multiplicity of analyses, results from similar studies, and other relevant evidence | 20. Discussion section (p10 of manuscript) |  |  |
| Generalisability | 21 | Discuss the generalisability (external validity) of the study results | 21. Discussion section (p10 of manuscript) |  |  |
| **Other Information** | | | | | |
| Funding | 22 | Give the source of funding and the role of the funders for the present study and, if applicable, for the original study on which the present article is based | 22. Acknowledgements section (p11 of manuscript) |  |  |
| Accessibility of protocol, raw data, and programming code |  | .. |  | RECORD 22.1: Authors should provide information on how to access any supplemental information such as the study protocol, raw data, or programming code. | 22.1. Statistical analysis section (p6 of manuscript) and data sharing section (p10 of manuscript) |

*Reference: Benchimol EI, Smeeth L, Guttmann A, Harron K, Moher D, Petersen I, Sørensen HT, von Elm E, Langan SM, the RECORD Working Committee. The REporting of studies Conducted using Observational Routinely-collected health Data (RECORD) Statement. *PLoS Medicine* 2015; in press. *Checklist is protected under Creative Commons Attribution ([CC BY](http://creativecommons.org/licenses/by/4.0/)) license.

# **Table S2. Developmental concerns in each part of analysis period, 13-15 month child health reviews and 27-30 month child health reviews**

|  | **13-15 Month Child Health Reviews** | | | | **27-30 Month Child Health Reviews** | | | |
| --- | --- | --- | --- | --- | --- | --- | --- | --- |
|  | **Total Analysis period** | **Pre**  **PHSM Period** | **During PHSM Period** | **Post**  **PHSM Period** | **Total Analysis period** | **Pre**  **PHSM Period** | **During PHSM Period** | **Post**  **PHSM Period** |
| **Any developmental concerns** | 186,265 (100%) | 49,131 (100%) | 54,898 (100%) | 82,236 (100%) | 186,766 (100%) | 50,461 (100%) | 55,889 (100%) | 80,416 (100%) |
| No | 166,216 (89.2%) | 44,227 (90.0%) | 49,709 (90.6%) | 72,280 (87.9%) | 158,713 (85.0%) | 43,820 (86.8%) | 47,887 (85.7%) | 67,006 (83.3%) |
| Yes | 20,049 (10.8%) | 4,904 (10.0%) | 5,189 (9.4%) | 9,956 (12.1%) | 28,053 (15.0%) | 6,641 (13.2%) | 8,002 (14.3%) | 13,410 (16.7%) |
| **Speech-language-communication developmental concerns** |  |  |  |  |  |  |  |  |
| No | 176,699 (94.9%) | 47,032 (95.7%) | 52,266 (95.2%) | 77,401 (94.1%) | 165,494 (88.6%) | 45,642 (90.5%) | 49,784 (89.1%) | 70,068 (87.1%) |
| Yes | 9,566 (5.1%) | 2,099 (4.3%) | 2,632 (4.8%) | 4,835 (5.9%) | 21,272 (11.4%) | 4,819 (9.5%) | 6,105 (10.9%) | 10,348 (12.9%) |
| **Problem solving developmental concerns** |  |  |  |  |  |  |  |  |
| No | 180,346 (96.8%) | 47,703 (97.1%) | 53,401 (97.3%) | 79,242 (96.4%) | 178,590 (95.6%) | 48,706 (96.5%) | 53,866 (96.4%) | 76,018 (94.5%) |
| Yes | 5,919 (3.2%) | 1,428 (2.9%) | 1,497 (2.7%) | 2,994 (3.6%) | 8,176 (4.4%) | 1,755 (3.5%) | 2,023 (3.6%) | 4,398 (5.5%) |
| **Gross motor developmental concerns** |  |  |  |  |  |  |  |  |
| No | 79,242 (96.4%) | 46,238 (94.1%) | 52,191 (95.1%) | 76,343 (92.8%) | 182,049 (97.5%) | 49,300 (97.7%) | 54,676 (97.8%) | 78,073 (97.1%) |
| Yes | 2,994 (6.1%) | 2,893 (5.9%) | 2,707 (4.9%) | 5,893 (7.2%) | 4,717 (2.5%) | 1,161 (2.3%) | 1,213 (2.2%) | 2,343 (2.9%) |
| **Personal-social developmental concerns** |  |  |  |  |  |  |  |  |
| No | 181,775 (97.6%) | 48,034 (97.8%) | 53,753 (97.9%) | 79,988 (97.3%) | 176,473 (94.5%) | 48,207 (95.5%) | 53,227 (95.2%) | 75,039 (93.3%) |
| Yes | 4,490 (2.4%) | 1,097 (2.2%) | 1,145 (2.1%) | 2,248 (2.7%) | 10,293 (5.5%) | 2,254 (4.5%) | 2,662 (4.8%) | 5,377 (6.7%) |
| **Emotional-behavioural developmental concerns** |  |  |  |  |  |  |  |  |
| No | 184,142 (98.9%) | 48,701 (99.1%) | 54,344 (99.0%) | 81,097 (98.6%) | 175,460 (93.9%) | 47,895 (94.9%) | 52,912 (94.7%) | 74,653 (92.8%) |
| Yes | 2,123 (1.1%) | 430 (0.9%) | 554 (1.0%) | 1,139 (1.4%) | 11,306 (6.1%) | 2,566 (5.1%) | 2,977 (5.3%) | 5,763 (7.2%) |
| **Fine motor developmental concerns** |  |  |  |  |  |  |  |  |
| No | 180,737 (97.0%) | 47,749 (97.2%) | 53,499 (97.5%) | 79,489 (96.7%) | 179,312 (96.0%) | 48,707 (96.5%) | 53,939 (96.5%) | 76,666 (95.3%) |
| Yes | 5,528 (3.0%) | 1,382 (2.8%) | 1,399 (2.5%) | 2,747 (3.3%) | 7,454 (4.0%) | 1,754 (3.5%) | 1,950 (3.5%) | 3,750 (4.7%) |

Notes: Data are N (%). The total analysis period was January 2019-August 2023, the pre PHSM period was January 2019-March 2020, the during PHSM period was March 2020-August 2021 and the post PHSM period was August 2021-August 2023

# **Model Assumption Checks**

**13-15 months – any developmental concerns**

Portmanteau test checking residuals resemble white noise

| Portmanteau (Q) statistic: 41.548 |
| --- |
| P value = 0.403 |

Kernel density plot showing model residuals with normal density overlaid


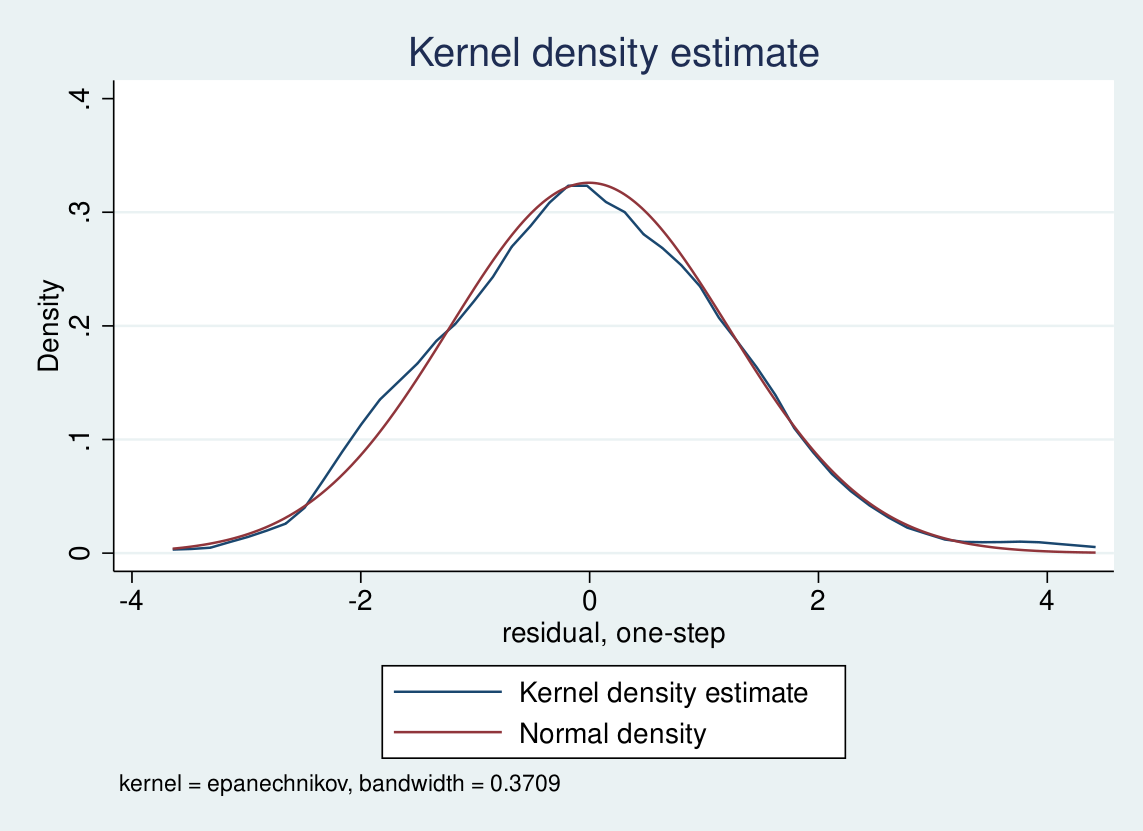


**13-15 months – speech-language-communication developmental concerns**

Portmanteau test checking residuals resemble white noise

| Portmanteau (Q) statistic: 30.964 |
| --- |
| P value = 0.847 |

Kernel density plot showing model residuals with normal density overlaid


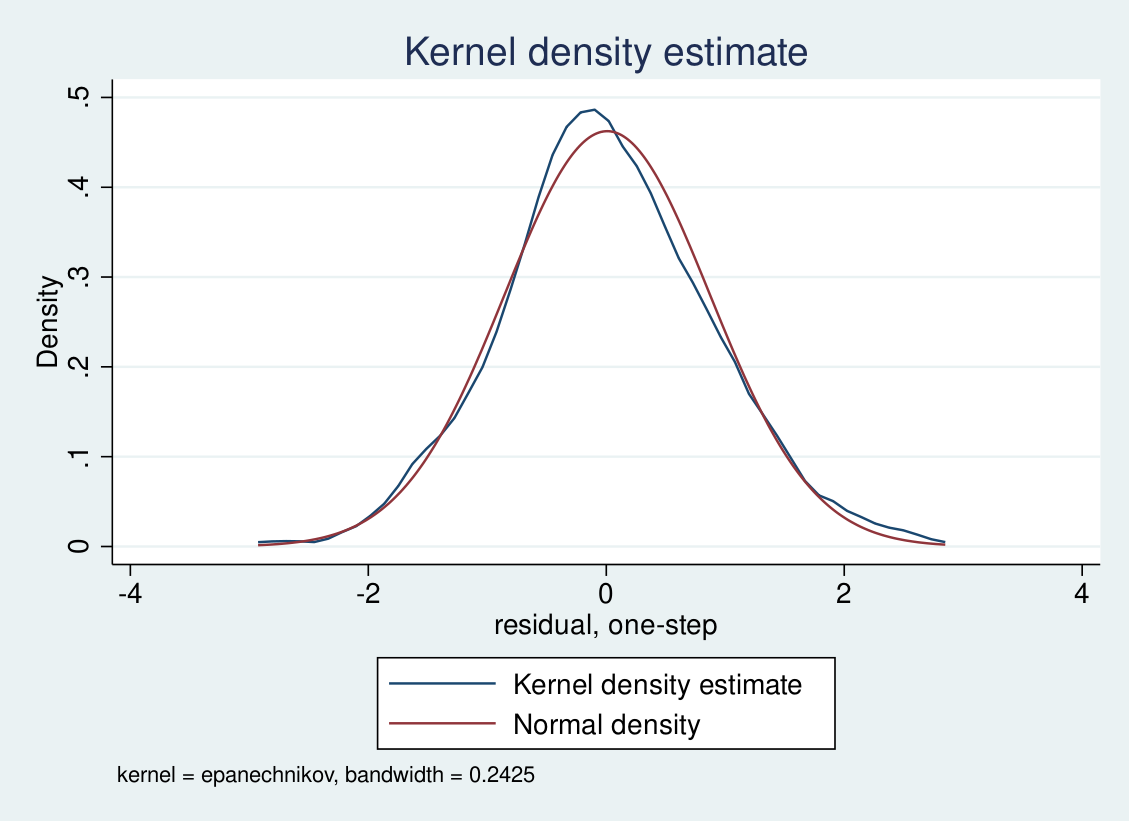


**13-15 months – problem solving developmental concerns**

Portmanteau test checking residuals resemble white noise

| Portmanteau (Q) statistic: 41.253 |
| --- |
| P value = 0.416 |

Kernel density plot showing model residuals with normal density overlaid


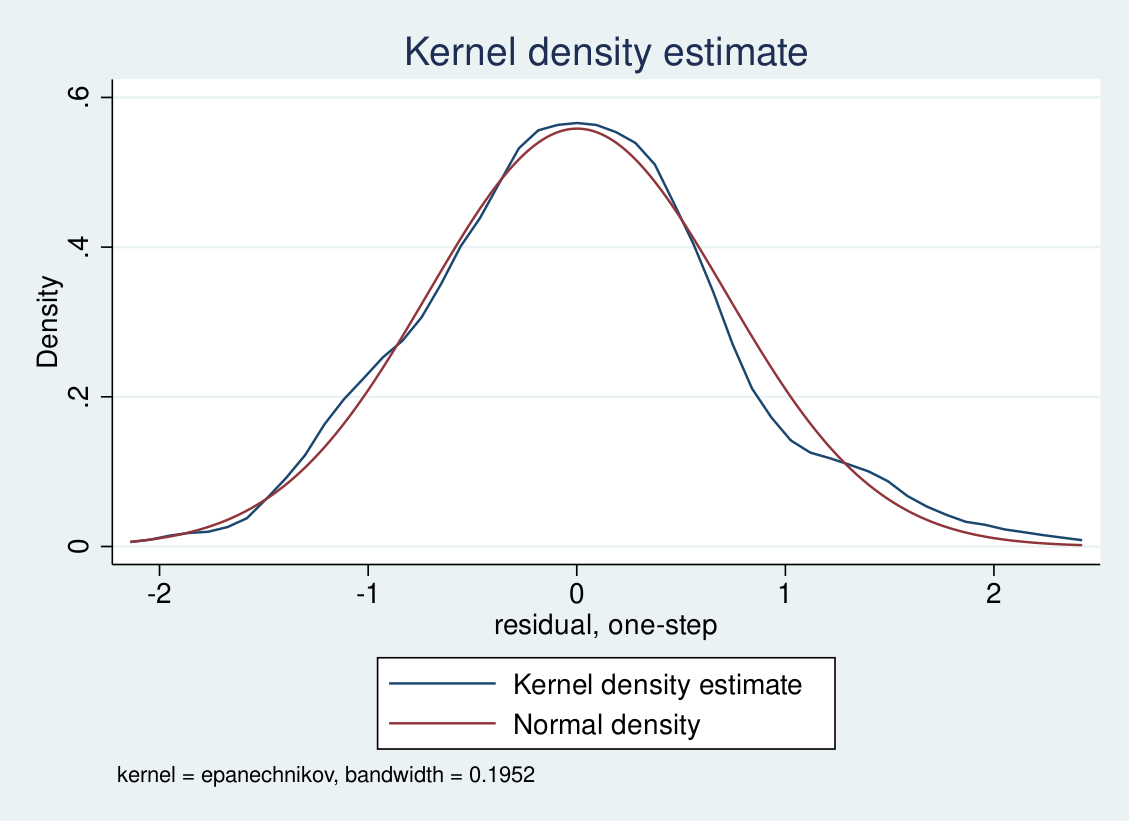


**13-15 months – gross motor developmental concerns**

Portmanteau test checking residuals resemble white noise

| Portmanteau (Q) statistic: 47.807 |
| --- |
| P value = 0.185 |

Kernel density plot showing model residuals with normal density overlaid


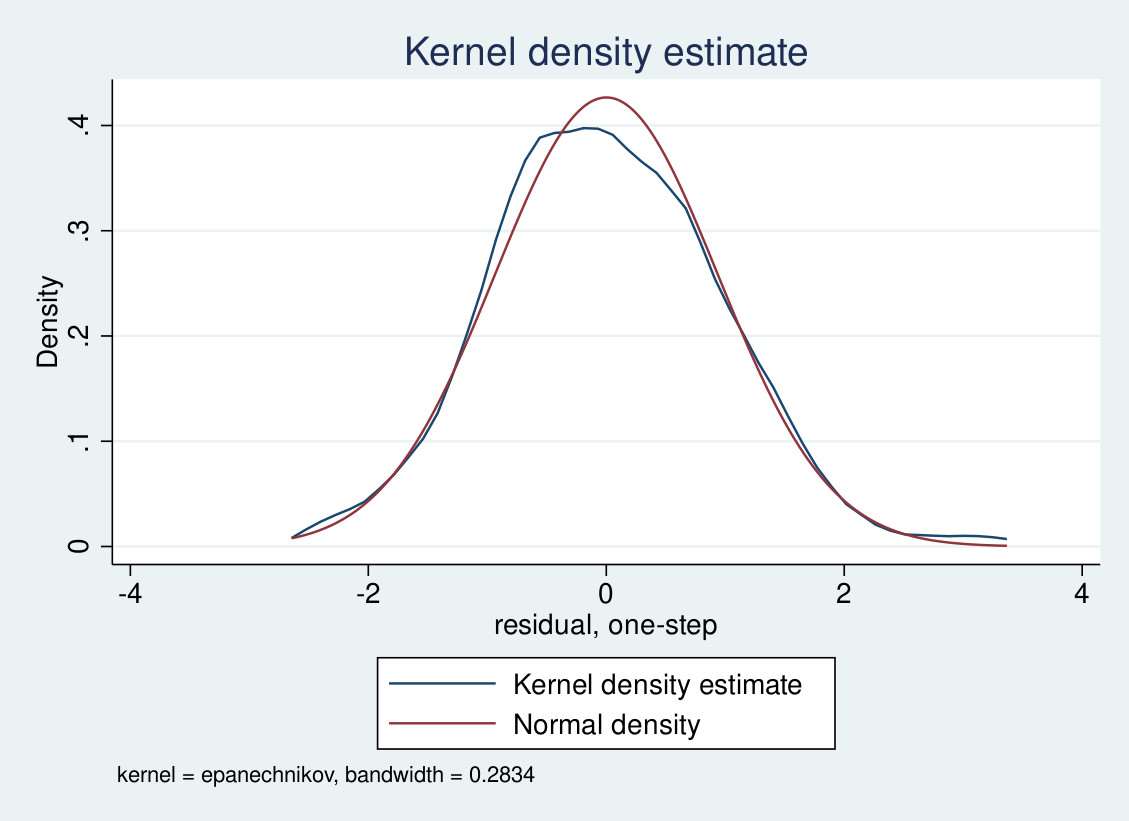


**13-15 months – personal-social developmental concerns**

Portmanteau test checking residuals resemble white noise

| Portmanteau (Q) statistic: 19.947 |
| --- |
| P value = 0.997 |

Kernel density plot showing model residuals with normal density overlaid


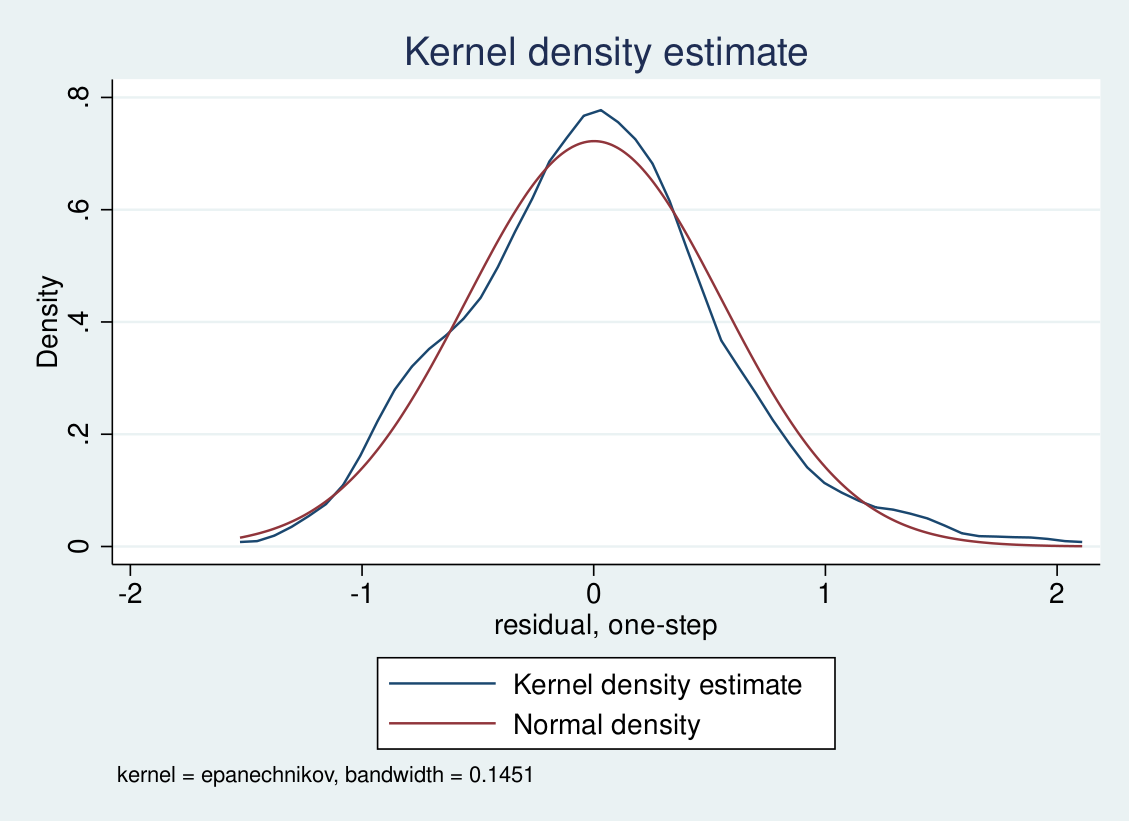


**13-15 months – emotional-behavioural developmental concerns**

Portmanteau test checking residuals resemble white noise

| Portmanteau (Q) statistic: 34.138 |
| --- |
| P value = 0.731 |

Kernel density plot showing model residuals with normal density overlaid


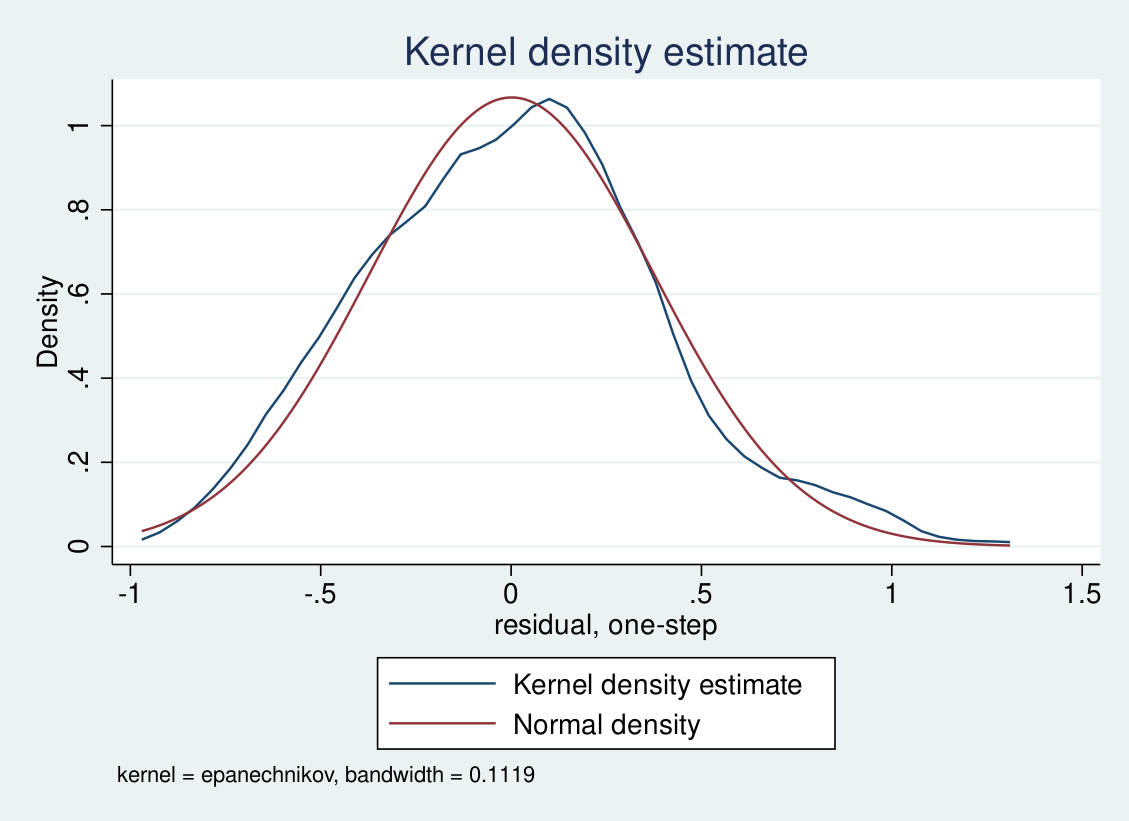


**13-15 months – fine motor developmental concerns**

Portmanteau test checking residuals resemble white noise

| Portmanteau (Q) statistic: 52.042 |
| --- |
| P value = 0.096 |

Kernel density plot showing model residuals with normal density overlaid


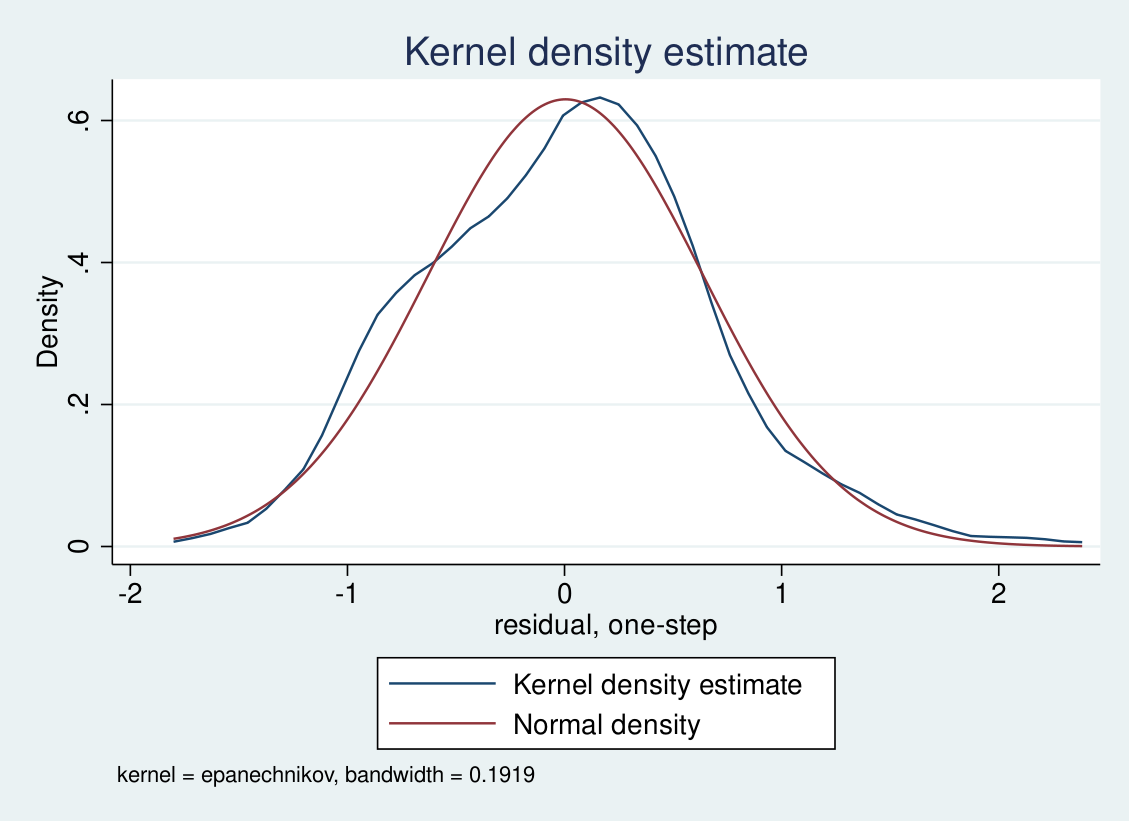


**27-30 months – any developmental concerns**

Portmanteau test checking residuals resemble white noise

| Portmanteau (Q) statistic: 26.606 |
| --- |
| P value = 0.949 |


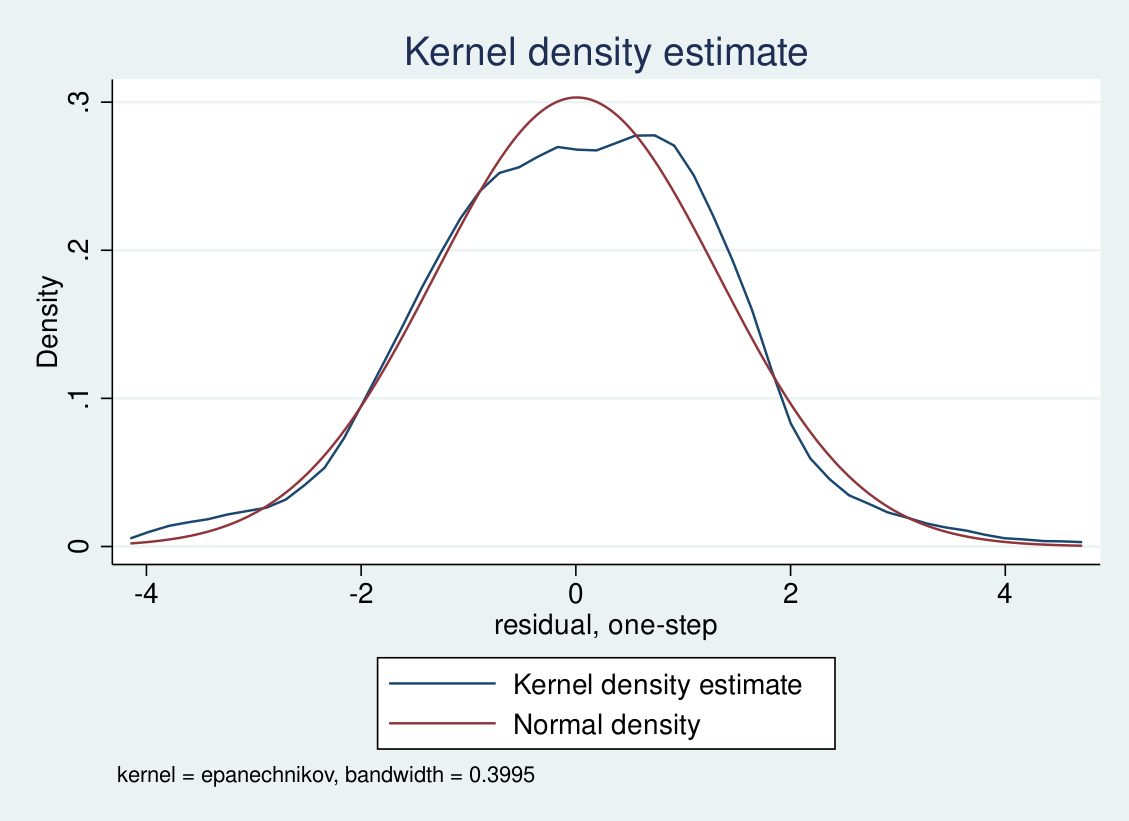
Kernel density plot showing model residuals with normal density overlaid

**27-30 months – speech-language-communication developmental concerns**

Portmanteau test checking residuals resemble white noise

| Portmanteau (Q) statistic: 42.710 |
| --- |
| P value = 0.356 |

Kernel density plot showing model residuals with normal density overlaid


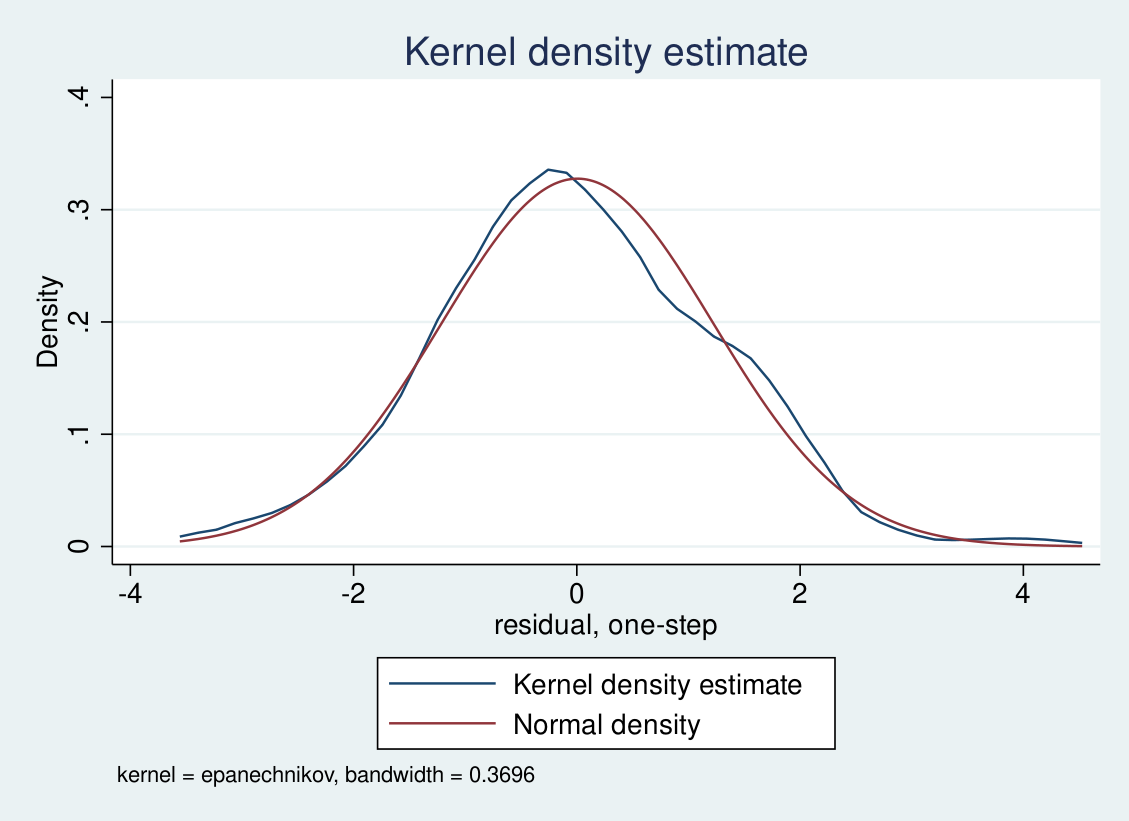


**27-30 months – problem solving developmental concerns**

Portmanteau test checking residuals resemble white noise

| Portmanteau (Q) statistic: 32.507 |
| --- |
| P value = 0.794 |

Kernel density plot showing model residuals with normal density overlaid


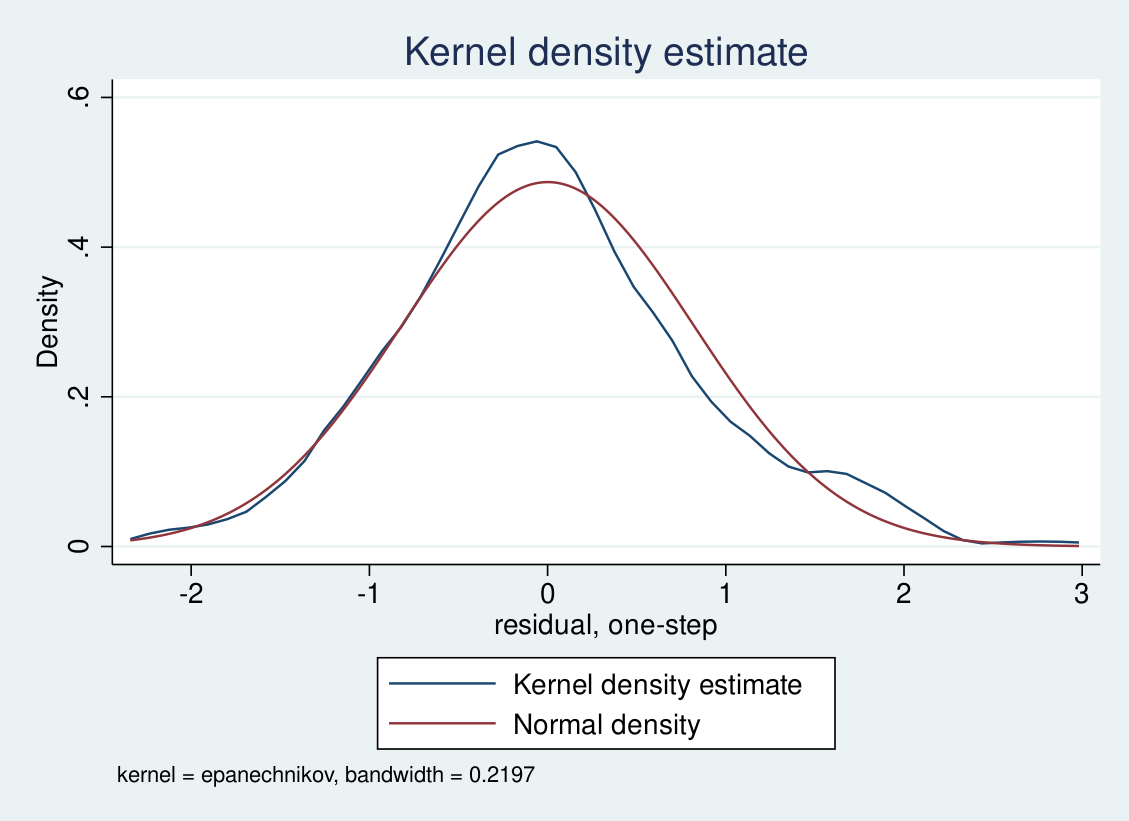


**27-30 months – gross motor developmental concerns**

Portmanteau test checking residuals resemble white noise

| Portmanteau (Q) statistic: 37.168 |
| --- |
| P value = 0.599 |

Kernel density plot showing model residuals with normal density overlaid


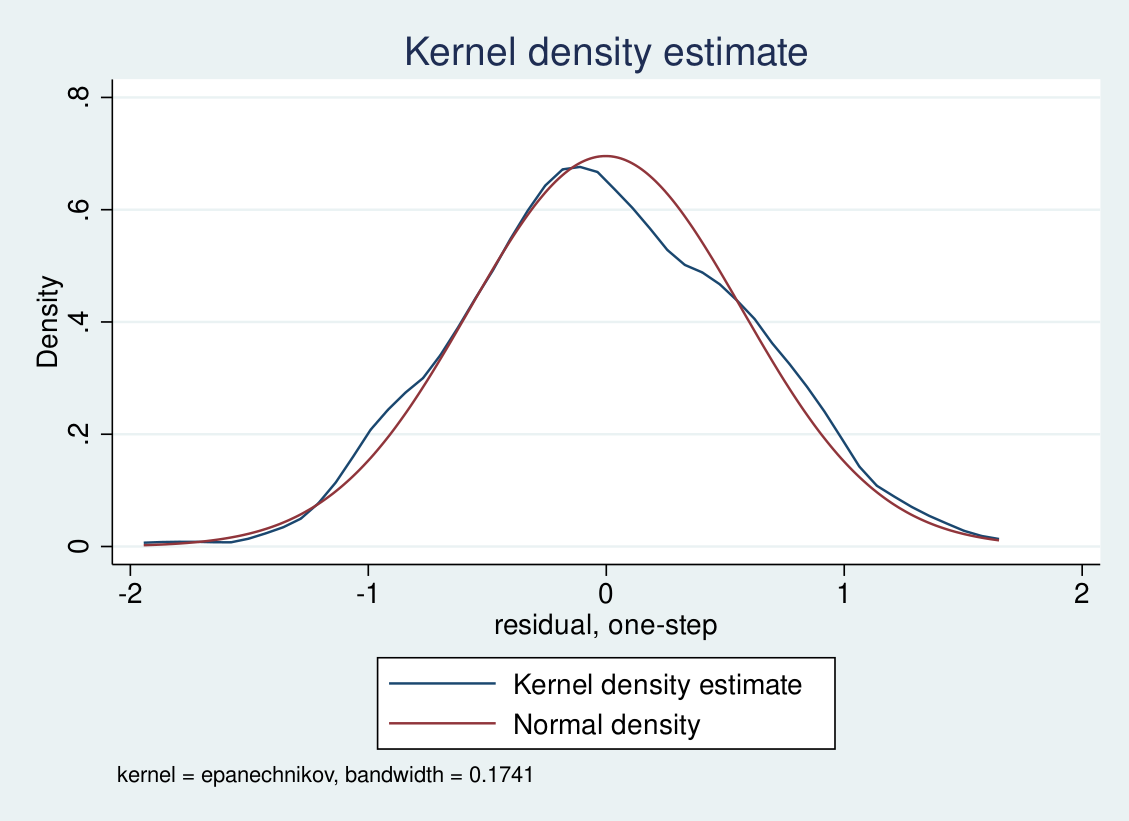


**27-30 months – personal-social developmental concerns**

Portmanteau test checking residuals resemble white noise

| Portmanteau (Q) statistic: 34.248 |
| --- |
| P value = 0.726 |

Kernel density plot showing model residuals with normal density overlaid


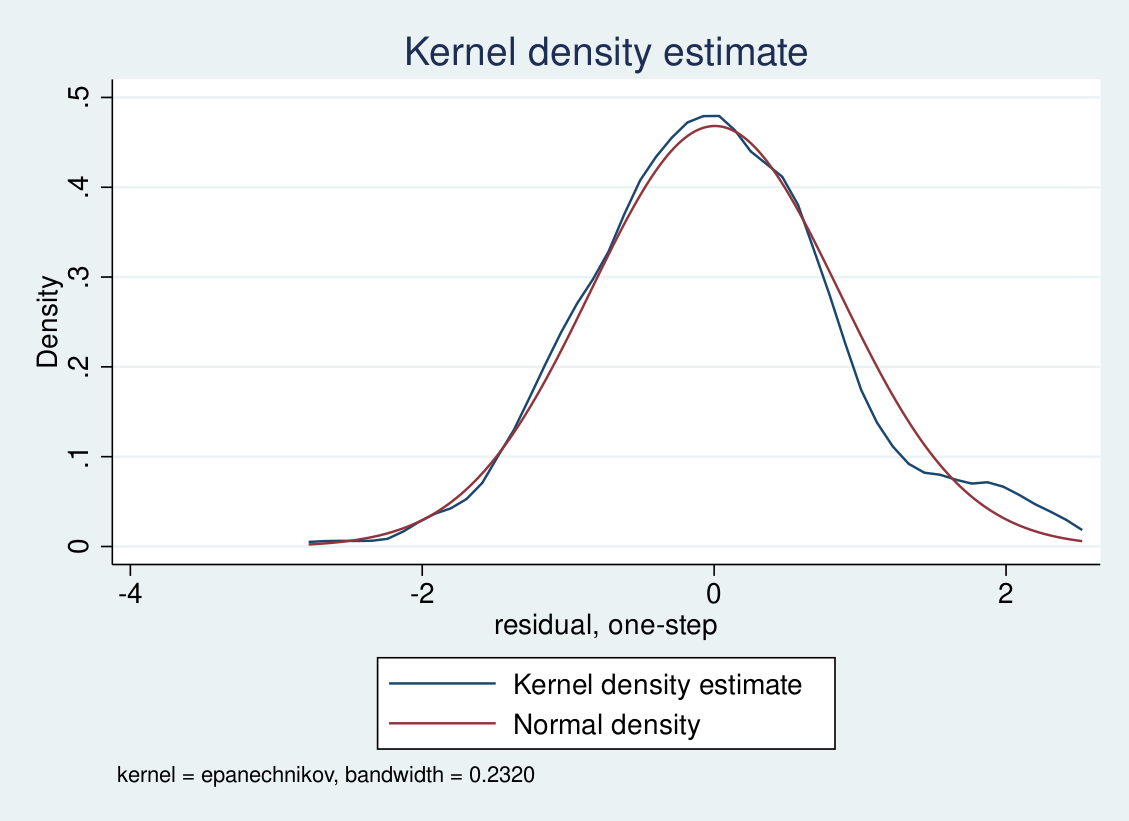


**27-30 months – emotional-behavioural developmental concerns**

Portmanteau test checking residuals resemble white noise

| Portmanteau (Q) statistic: 32.845 |
| --- |
| P value = 0.782 |

Kernel density plot showing model residuals with normal density overlaid


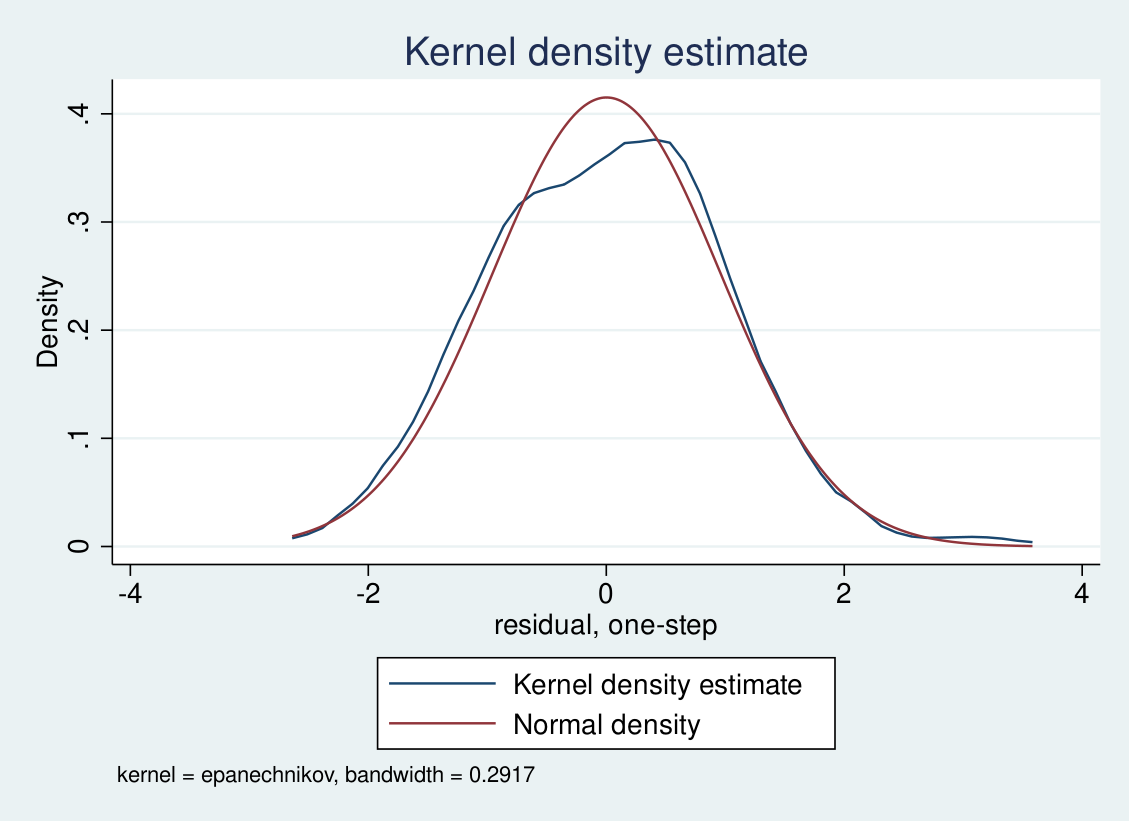


**27-30 months – fine motor developmental concerns**

Portmanteau test checking residuals resemble white noise

| Portmanteau (Q) statistic: 29.321 |
| --- |
| P value = 0.893 |

Kernel density plot showing model residuals with normal density overlaid


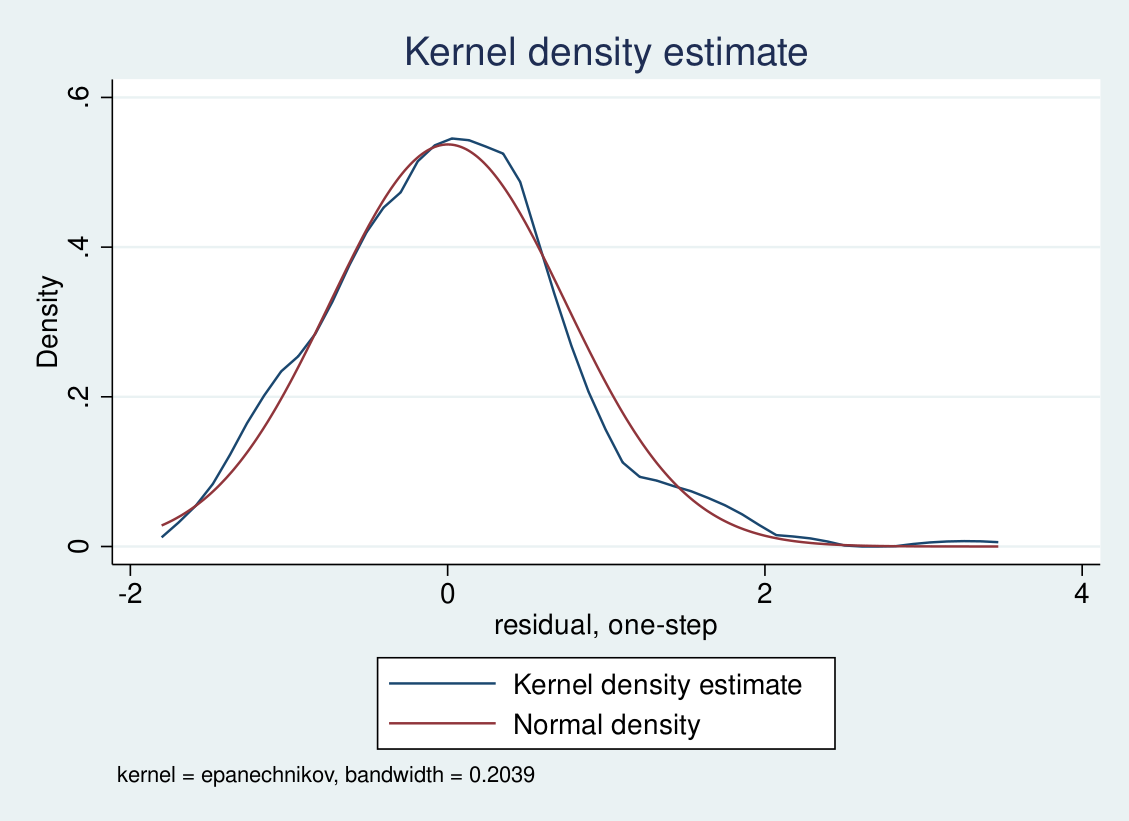


# **Table S3. Sensitivity analysis (narrower analysis period of August 2019-August 2023) showing associations between COVID-19 PHSM introduction, COVID-19 PHSM removal and slope changes in the weekly proportion of children with developmental concerns identified at 13-15 month child health reviews**

|  | **13-15 month child health reviews,**  **slope change B (95% CI)** | | | |
| --- | --- | --- | --- | --- |
|  | **PHSM introduction** | **PHSM removal** | **Constant** | **AR terms** |
| **Any developmental concerns** | 0.058  (0.007, 0.109) | -0.006  (-0.022, 0.011) | 93.87  (-40.721, 228.452) | L3: 0.238  (0.089, 0.388)  L36: -0.262  (-0.418, -0.105) |
| **Speech-language-communication concerns** | 0.004  (-0.033, 0.041 | -0.008  (-0.017, 0.002) | -35.532  (-139.537, 68.474) | L29: -0.229  (-0.39, -0.071) |
| **Problem solving concerns** | 0.029  (-0.003, 0.061) | -0.003  (-0.013, 0.007) | 57.458  (-26.714, 141.631) | L1: 0.178  (0.034, 0.323) |
| **Gross motor concerns** | 0.570  (0.020, 0.094) | 0.010  (-0.004, 0.025) | 133.477  (35.356, 231.597) | L3: 0.161  (0.003, 0.320)  L5: 0.165  (0.012, 0.317) |
| **Personal-social concerns** | 0.134  (-0.011, 0.038) | 0.001  (-0.005, 0.007) | 24.272  (-45.666, 94.211) | L29: -0.137  (-0.317, 0.044) |
| **Emotional-behavioural concerns** | -0.004  (-0.020, 0.012) | 0.003  (-0.001, 0.007) | -18.791  (-62.302, 24.721) | L20: -0.067  (-0.229, 0.095) |
| **Fine motor concerns** | 0.005  (-0.028, 0.038) | 0.007  (-0.004, 0.017) | 4.329  (-87.303, 95.962) | L4: 0.165  (0.015, 0.315)  L7: 0.205  (0.072, 0.338) |

ARIMA model estimates adjust for autocorrelation and trend. PHSM introduction was the 12^th^ week of 2020, reflecting the fact that strict lockdown measures were first introduced in Scotland on 23^rd^ March 2020. PHSM removal was the 32^nd^ week of 2021, reflecting the fact that all remaining social distancing measures were removed on 9^th^ August 2021 (this is used as an indicator of the general ending of PHSM, although some restrictions were temporarily reintroduced in December-January 2021). B = coefficient, 95% CI = 95% confidence intervals. Results for the AR terms included in each model are provided next to main results, with L referring to the number of lags. These were selected following an iterative process involving autocorrelation function and partial autocorrelation function plots and model fit statistics.

# **Table S4. Sensitivity analysis (narrower analysis period of May 2019-August 2023) showing associations between COVID-19 PHSM introduction, COVID-19 PHSM removal and slope changes in the weekly proportion of children with developmental concerns identified at 27-30 month child health reviews**

|  | **Slope change B (95% CI)** | | | |
| --- | --- | --- | --- | --- |
|  | **PHSM introduction** | **PHSM removal** | **Constant** | **AR terms** |
| **Any developmental concerns** | 0.119  (0.085, 0.152) | -0.074  (-0.085, -0.059) | 167.876  (82.161, 253.591) | L1: 0.128  (-0.007, 0.262) |
| **Problem solving concerns** | 0.060  (0.034, 0.086) | -0.018  (-0.029, -0.008) | 94.950  (29.681, 160.218) | L1: 0.121  (-0.051, 0.294) |

ARIMA model estimates adjust for autocorrelation and trend. PHSM introduction was the 12^th^ week of 2020, reflecting the fact that strict lockdown measures were first introduced in Scotland on 23^rd^ March 2020. PHSM removal was the 32^nd^ week of 2021, reflecting the fact that all remaining social distancing measures were removed on 9^th^ August 2021 (this is used as an indicator of the general ending of PHSM, although some restrictions were temporarily reintroduced in December-January 2021). B = coefficient, 95% CI = 95% confidence intervals. Results for the AR terms included in each model are provided next to main results, with L referring to the number of lags. These were selected following an iterative process involving autocorrelation function and partial autocorrelation function plots and model fit statistics.

# **Table S5. Sensitivity analysis (Greater Glasgow & Clyde health board excluded) showing associations between COVID-19 PHSM introduction, COVID-19 PHSM removal and slope changes in the weekly proportion of children with developmental concerns identified at 13-15 month and 27-30 month child health reviews**

|  | **13-15 month child health reviews,**  **slope change B (95% CI)** | | | | **27-30 month child health reviews,**  **slope change B (95% CI)** | | | |
| --- | --- | --- | --- | --- | --- | --- | --- | --- |
|  | **PHSM introduction** | **PHSM removal** | **Constant** | **AR terms** | **PHSM introduction** | **PHSM removal** | **Constant** | **AR terms** |
| **Any developmental concerns** | 0.082  (0.054, 0.110) | -0.032  (-0.050, -0.014) | 123.372  (64.555, 182.189) | L3: 0.164  (0.014, 0.315) | 0.090  (0.064, 0.116) | -0.067  (-0.085, -0.049) | 103.823  (51.986, 155.661) | L2: 0.185  (0.055, 0.316) |
| **Speech-language-communication concerns** | 0.029  (0.011, 0.048) | -0.025  (-0.037, -0.013) | 11.928  (-28.053, 51.910) | L11: 0.171  (0.030, 0.313) | 0.082  (0.063, 0.101) | -0.064  (-0.077, -0.052) | 83.189  (45.731, 120.646) | L14: -0.11  (-0.250, 0.029) |
| **Problem solving concerns** | 0.032  (0.013, 0.051) | -0.014  (-0.024, -0.004) | 47.153  (5.966, 88.340) | L1: 0.124  (-0.011, 0.259) | 0.053  (0.035, 0.070) | -0.033  (-0.044, -0.022) | 63.608  (26.334, 100.882) | L1: 0.155  (-0.004, 0.314) |
| **Gross motor concerns** | 0.050  (0.031, 0.068) | 0.003  (-0.010, 0.016) | 94.145  (53.777, 134.514) | L18: -0.013  (-0.013, 0.265) | 0.018  (0.003, 0.03) | -0.005  (-0.014, 0.002) | 29.355  (-2.731, 61.441) | L4: 0.131  (-0.011, 0.273) |
| **Personal-social concerns** | 0.018  (0.007, 0.029) | -0.004  (0.010, 0.002) | 30.519  (5.738, 55.299) | L29: -0.152  (-0.304, -0.000) | 0.059  (0.040, 0.079) | -0.036  (-0.050, -0.023) | 77.864  (36.261, 119.466) | L1: 0.278  (0.124, 0.431) |
| **Emotional-behavioural concerns** | 0.004  (-0.006, 0.002) | -0.002  (-0.006, 0.002) | -1.881  (-15.264, 11.502) | L5: -0.152  (-0.294, -0.010) | 0.046  (0.026, 0.067) | -0.025  (-0.038, -0.011) | 61.204  (18.229, 104.180) | L1: 0.220  (0.093, 0.346) |
| **Fine motor concerns** | 0.023  (0.010, 0.035) | -0.004  (-0.011, 0.003) | 37.702  (10.531, 64.874) | L15: -0.136  (-0.265, -0.008) | 0.041  (0.025, 0.056) | -0.026  (-0.036, -0.017) | 52.487  (20.660, 84.313) | L1: 0.166  (0.028, 0.304)  L10: -0.157  (-0.292, -0.022) |

ARIMA model estimates adjust for autocorrelation and trend. PHSM introduction was the 12^th^ week of 2020, reflecting the fact that strict lockdown measures were first introduced in Scotland on 23^rd^ March 2020. PHSM removal was the 32^nd^ week of 2021, reflecting the fact that all remaining social distancing measures were removed on 9^th^ August 2021 (this is used as an indicator of the general ending of PHSM, although some restrictions were temporarily reintroduced in December-January 2021). B = coefficient, 95% CI = 95% confidence intervals. Results for the AR terms included in each model are provided next to main results, with L referring to the number of lags. These were selected following an iterative process involving autocorrelation function and partial autocorrelation function plots and model fit statistics.

# **Table S6. Sensitivity analysis (narrower analysis period of January 2019-December 2022) showing associations between COVID-19 PHSM introduction, COVID-19 PHSM removal and slope changes in the weekly proportion of children with developmental concerns identified at 13-15 month child health reviews**

|  | **13-15 month child health reviews,**  **slope change B (95% CI)** | | | |
| --- | --- | --- | --- | --- |
|  | **PHSM introduction** | **PHSM removal** | **Constant** | **AR terms** |
| **Any developmental concerns** | 0.099  (0.076, 0.122) | -0.031  (-0.052, -0.011) | 192.247  (147.095, 237.400) | L3: 0.167  (0.019, 0.315) |
| **Speech-language-communication concerns** | 0.042  (0.024, 0.059) | -0.026  (-0.043, -0.010) | 57.044  (22.190, 91.899) | L4: 0.168  (0.016, 0.320)  L11: 0.172  (0.015, 0.329) |
| **Problem solving concerns** | 0.037  (0.026, 0.47) | -0.016  (-0.025, -0.008) | 68.533  (47.166, 89.900) | L9: -0.154  (-0.298, -0.010)  L32: -0.178  (-0.34, -0.019) |
| **Gross motor concerns** | 0.060  (0.044, 0.076) | 0.000  (-0.015, 0.015) | 133.704  (100.961, 166.447) | L3: 0.153  (-0.017, 0.323) |
| **Personal-social concerns** | 0.022  (0.015, 0.030) | -0.006  (-0.012, 0.001) | 43.836  (27.257, 60.416) | L21: -0.164  (-0.302, -0.027)  L34: -0.180  (-0.313, -0.031) |
| **Emotional-behavioural concerns** | 0.005  (-0.002, 0.012) | -0.001  (-0.007, 0.005) | 3.545  (-10.463, 17.553) | L3: 0.153  (0.010, 17.553) |
| **Fine motor concerns** | 0.030  (0.021, 0.039) | -0.009  (-0.016, -0.001) | 62.076  (43.572, 80.580) | L2: -0.151  (-0.304, 0.001)  L37: -0.190  (-0.32, -0.055) |

ARIMA model estimates adjust for autocorrelation and trend. PHSM introduction was the 12^th^ week of 2020, reflecting the fact that strict lockdown measures were first introduced in Scotland on 23^rd^ March 2020. PHSM removal was the 32^nd^ week of 2021, reflecting the fact that all remaining social distancing measures were removed on 9^th^ August 2021 (this is used as an indicator of the general ending of PHSM, although some restrictions were temporarily reintroduced in December-January 2021). B = coefficient, 95% CI = 95% confidence intervals. Results for the AR terms included in each model are provided next to main results, with L referring to the number of lags. These were selected following an iterative process involving autocorrelation function and partial autocorrelation function plots and model fit statistics.
